# Biobased Poly(ethylene furanoate) Polyester/TiO_2_ Supported Nanocomposites as Effective Photocatalysts for Anti-inflammatory/Analgesic Drugs

**DOI:** 10.3390/molecules24030564

**Published:** 2019-02-04

**Authors:** Anastasia Koltsakidou, Zoi Terzopoulou, George Z. Kyzas, Dimitrios N. Bikiaris, Dimitra A. Lambropoulou

**Affiliations:** 1 Laboratory of Environmental Pollution Control, Department of Chemistry, Aristotle University of Thessaloniki, GR–541 24 Thessaloniki, Greece; akoltsakidou@gmail.com; 2 Laboratory of Polymer Chemistry and Technology, Department of Chemistry, Aristotle University of Thessaloniki, GR-541 24 Thessaloniki, Greece; terzoe@gmail.com (Z.T.), dbic@chem.auth.gr (D.N.B.); 3 Hephaestus Advanced Laboratory, Eastern Macedonia and Thrace Institute of Technology, GR-654 04 Kavala, Greece; kyzas@teiemt.gr

**Keywords:** poly(ethylene furanoate), polyester, titanium dioxide, nanocomposites, pharmaceuticals, photocatalysis

## Abstract

In the present study, polymer supported nanocomposites, consisting of bio-based poly(ethylene furanoate) polyester and TiO_2_ nanoparticles, were prepared and evaluated as effective photocatalysts for anti-inflammatory/analgesic drug removal. Nanocomposites were prepared by the solvent evaporation method containing 5, 10, 15, and 20 wt% TiO_2_ and characterized using Fourier Transform Infrared spectroscopy (FTIR), differential scanning calorimetry (DSC), wide-angle X-ray diffraction (WAXD), thermogravimetric analysis (TGA), and scanning electron microscopy (SEM). Thin films of them have been prepared by the melt press and optimization of the photocatalytic procedure was conducted for the most efficient synthesized photocatalyst. Finally, mineralization was evaluated by means of Total organic carbon (TOC) reduction and ion release, while the transformation products (TPs) generated during the photocatalytic procedure were identified by high-resolution mass spectrometry.

## 1. Introduction

During the last decades, photocatalysis is used for the treatment of a variety of pollutants such as dyes, pesticides pharmaceuticals, and various endocrine disrupting compounds [1,2,3,4]. Titanium dioxide has been reported as one of the most efficient photocatalysts, mostly because of its low cost, the high chemical, photo, and biological stability and high catalytic activity. Apart from that, development of multifunctional TiO_2_-based materials for biomedical applications were extensively reported in literature [5,6,7,8]. Titanium dioxide is commercially available in the form of powder that shows the greatest surface area and efficiency. However, when applied to wastewater in the form of powder, it suffers from the following drawbacks: Low light utilization efficiency of suspended photocatalyst, due to the attenuation loss suffered by light rays [9]; requirement of post-treatment recovery, that is both time and money consuming, and also leads to the loss of catalyst [10]; and possible cause of un-favorable human health problems associated with the mobility of the powder form [11].

In order to overcome all the aforementioned drawbacks, continuous efforts are being made to support TiO_2_ on various substrates. Membranes that are commonly used to purify water and to separate TiO_2_ particles from treated water are an ideal material to support TiO_2_. Since Anderson et al. introduced the photocatalysis by immobilizing TiO_2_ in a membrane [12], many scientists have applied it to both organic [13,14,15] and inorganic membranes [16]. Polymers such as polyamide, polysulfone, polyethersulfone, polyvinylidene fluoride, polypropylene, polyacrylonitrile, and cellulose acetate have been used as substrates for TiO_2_ based photocatalysts [17]. Moreover, the importance of immobilizing TiO_2_ in a polymer matrix can also be noted for different important applications, as for example, in hybrid photovoltaics [18]. Due to the ongoing progress in hybrid solar cells, the reached efficiency values become more and more competitive with conventional photovoltaics. The morphology of the active layer turned out to be very important for these devices. Mainly titania (TiO_2_) of different morphologies is used as a major component in such hybrid solar cells. In addition to research focusing on increasing device efficiencies, the development of alternative, more environmentally friendly hybrid solar cell fabrication routes is interesting.

Polymer support appears to be highly suitable for TiO_2_ due to a series of advantages. Firstly, polymers are innoxious materials, chemically inert and mechanically stable with high durability [19]. They can offer pre-concentration of the organic pollutants on their surface, increasing the efficiency of adsorption and subsequent oxidation, due to their hydrophobic nature [19]. Furthermore, they are inexpensive, readily available with thermos-softening properties, which increase the ease of coating TiO_2_ on them by simple thermal treatment methods [20]. Finally, many of them have high UV-resistance, hence they do not undergo oxidation readily [21].

Bio-based polymers that are either biodegradable or at least partly bio-based, constitute one of the fastest growing markets [22,23,24,25,26,27,28], due to the great concern about the depletion of fossil resources, as well as the growing accumulation of plastic waste in the environment. During the last years, the production of furan derivatives from sugars has become exciting in chemistry and in catalysis studies, because it aids one of the major routes for achieving sustainable energy supply and chemicals production. 5-HMF, 2,5-FDCA, and 2,5-dimethylfuran (2,5-DMF) have been called the “sleeping giants” of renewable intermediate chemicals and can be produced from biomass after its fermentation [29,30]. FDCA is considered the bio-based equivalent of terepthalic acid, therefore its polyester poly(ethylene furanoate) (PEF) is very promising as the bio-based alternative of poly(ethylene terephthalate) (PET) [31].

Since PEF is expected to be the bio-based polyester that will gradually replace its fossil-based counterpart PET, studying its properties and possible uses is a fast-developing research interest area. The furan ring provides PEF higher polarity, making it more compatible with TiO_2_ nanoparticles and therefore more suitable as a support polymer for TiO_2_ photocatalysts. 

The usual methods of supporting TiO_2_ on polymers are sol-gel and sputtering, however sol-gel techniques result in photocatalysts with amorphous TiO_2_, instead of the preferred anatase form, and require post-deposition thermal treatment in temperatures above 300 °C in order to recrystallize it [32]. Another possible approach for the support of TiO_2_ on polymers is solution mixing, which is an inexpensive technique that occurs in inert conditions and does not affect the crystal structure of anatase. Additionally, in comparison with sputtering or hot-pressing methods, it allows the polymeric substrate to form stronger interactions with the TiO_2_ nanoparticles. 

In the context of development environmentally friendly materials and based on the fact that the furan ring polarity makes the PEF a suitable substrate for the immobilization of TiO_2_, the aim of this study was the synthesis of PEF/nTiO_2_ nanocomposites, as a first stage of membrane PEF/TiO_2_ synthesis investigation, containing various concentrations (wt%) of TiO_2_. Additionally, the main objective of this study was to assess the application of the promising PEF/TiO_2_ membrane configuration in the photocatalytic treatment of commonly detected contaminants like Non-Steroidal Anti-Inflammatory Drugs (NSAIDs). Among all the human medicines, NSAIDs are one of the most frequently recommended pain killer medicines. Large amounts of these painkillers are prescribed in human medical care, but more often, they are sold without prescription as so-called ‘over-the-counter’ drugs. Residues of NSAIDs are usually present in surface water and ground water or even in drinking water [33,34,35]. Despite this, they have been detected in wastewater treatment plants, sewage sludge, and agricultural soils due to application of manure contaminated with pharmaceuticals, irrigation with non-reclaimed or treated wastewater [36]. Therefore, removal of NSAIDs from water systems is necessary considering its potentially harmful impacts on the environment. In the present study, a mixture of such extensively found contaminants in the aquatic system such as Ibuprofen (IBU), Diclofenac (DCF) and Acetaminophen (ACM) was selected. These NSAIDs are among the most frequently utilized drugs and the NORMAN experts have included them in the list of the ‘emerging contaminants’.

Further emphasis was given to optimize the photocatalytic procedure using different parameters (drug concentration, catalyst concentration and pH). To the best of our knowledge, this is the first study regarding the synthesis of PEF/TiO_2_ nanocomposites, evaluation of its properties, and its application for the photocatalytic treatment of pharmaceuticals.

## 2. Results and Discussion

### 2.1. Synthesis and Characterization of PEF/TiO_2_ Photocatalysts

**Scheme 1:**
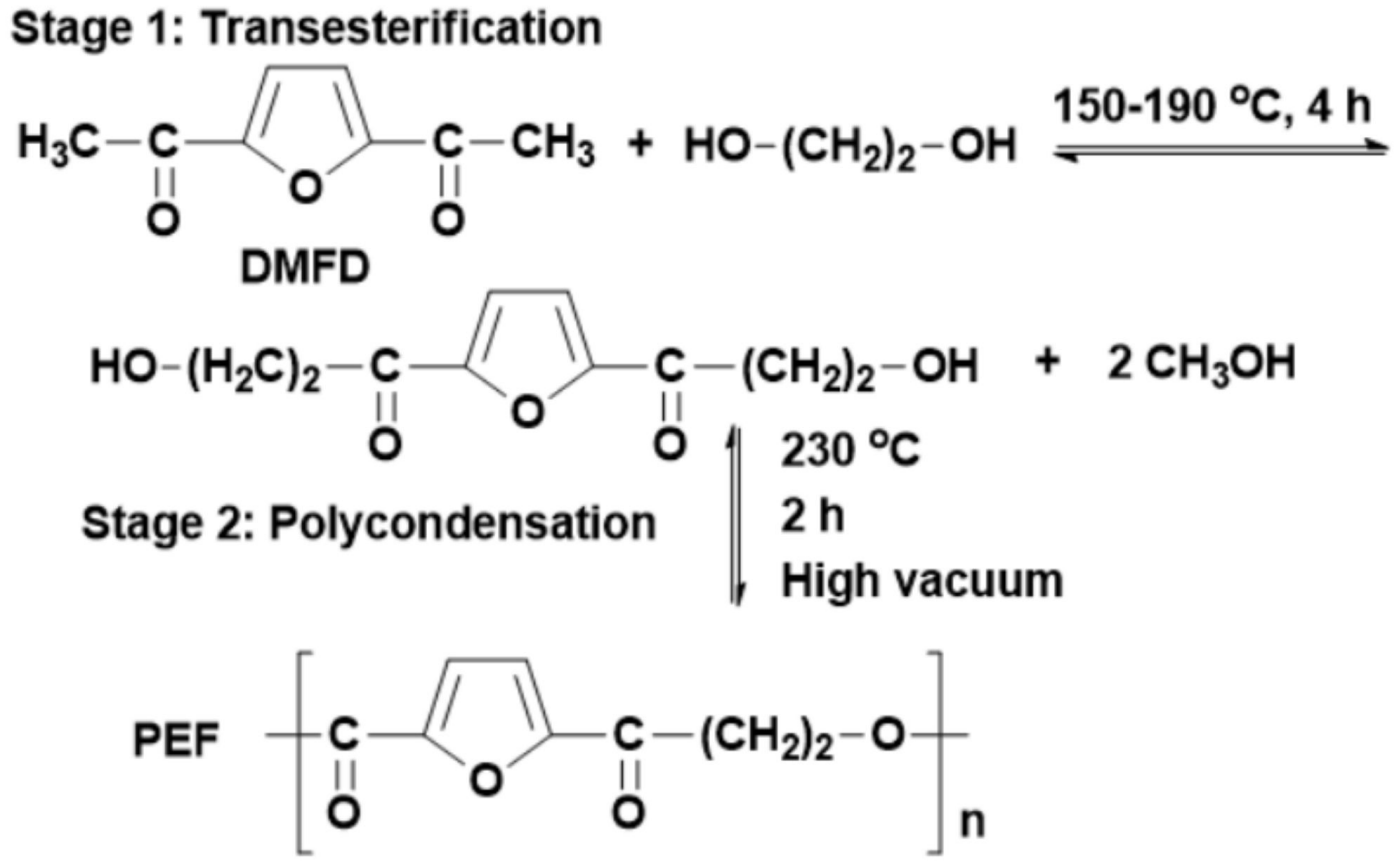
Synthesis of PEF with the two-step polycondensation reaction.

PEF was synthesized from DMFD by the two-step melt polycondensation method with the use of TBT catalyst (Scheme 1). The final product had a IV value of 0.45 dL g^−1^, which is within the values reported for the same catalyst in previous studies [37,38] and what was found by GPC PEF has M_n_ = 11200 g mol^−1^. Since the polyester exhibited a yellow discoloration, a purification process took place in order to remove impurities and unreacted monomers. For this reason, PEF was dissolved in a mixture of trifluoroacetic acid/chloroform 1/4 *v*/*v* and precipitated in cold methanol. The product obtained after the purification was a white powder, which means that some decomposition products formed during synthesis of PEF have been removed during this treatment [39].

**Figure 1:**
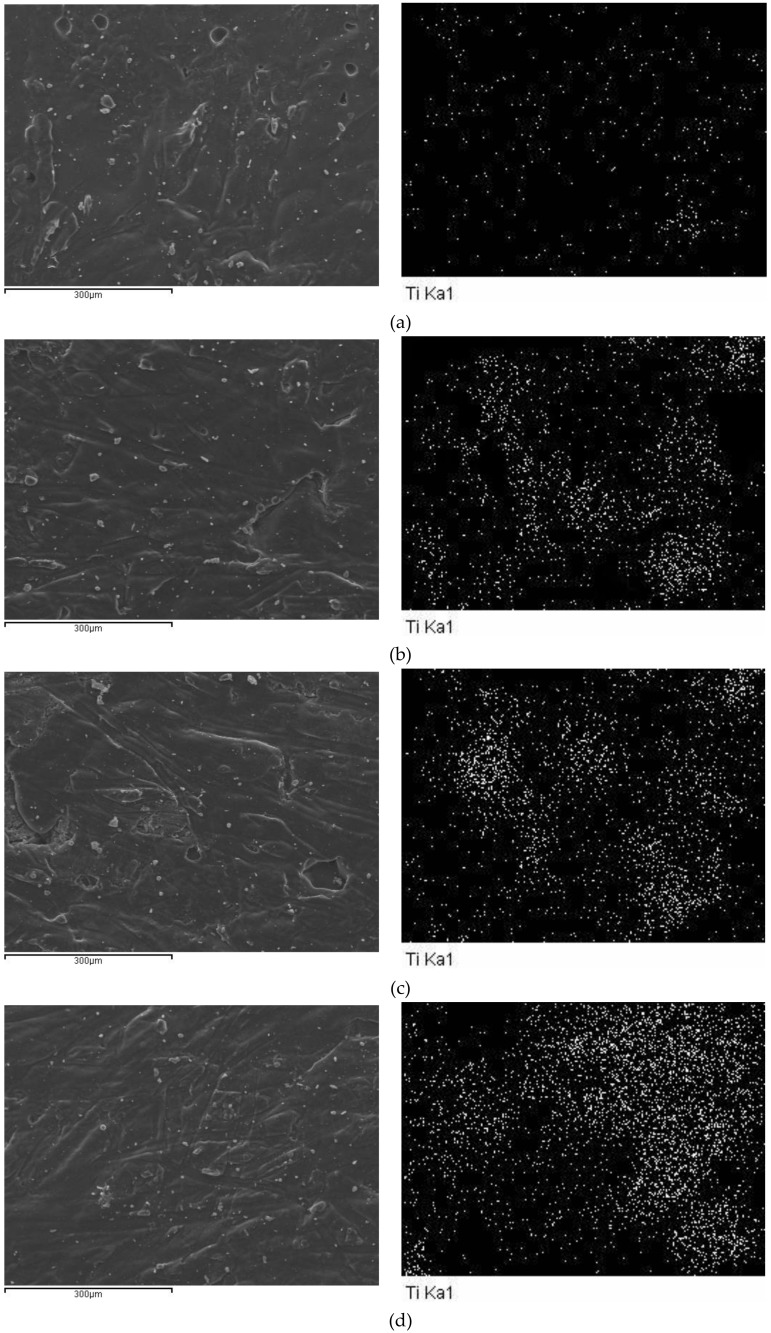
SEM micrographs and Ti mapping of (**a**) PEF/TiO_2_ 5%, (**b**) PEF/TiO_2_ 10%, (**c**) PEF/TiO_2_ 15%, and (**d**) PEF/TiO_2_ 20%.

TiO_2_ was supported on the synthesized PEF by preparing PEF/TiO_2_ nanocomposites with the solution mixing method firstly and after melt pressing thin films (1 mm) in final TiO_2_ concentrations 5, 10, 15, and 20 wt% have been prepared. The morphology and dispersion of the nanocomposites powder was studied by SEM and EDX element mapping. SEM micrographs and their corresponding Ti maps are presented in Figure 1. The respective mapping images reveal satisfactory dispersion of the nanoparticles into the polymer matrix, and the density of the maps increases while increasing TiO_2_ concentration. This suggests that PEF is capable of supporting TiO_2_ quantities up to 20 wt%.

**Figure 2:**
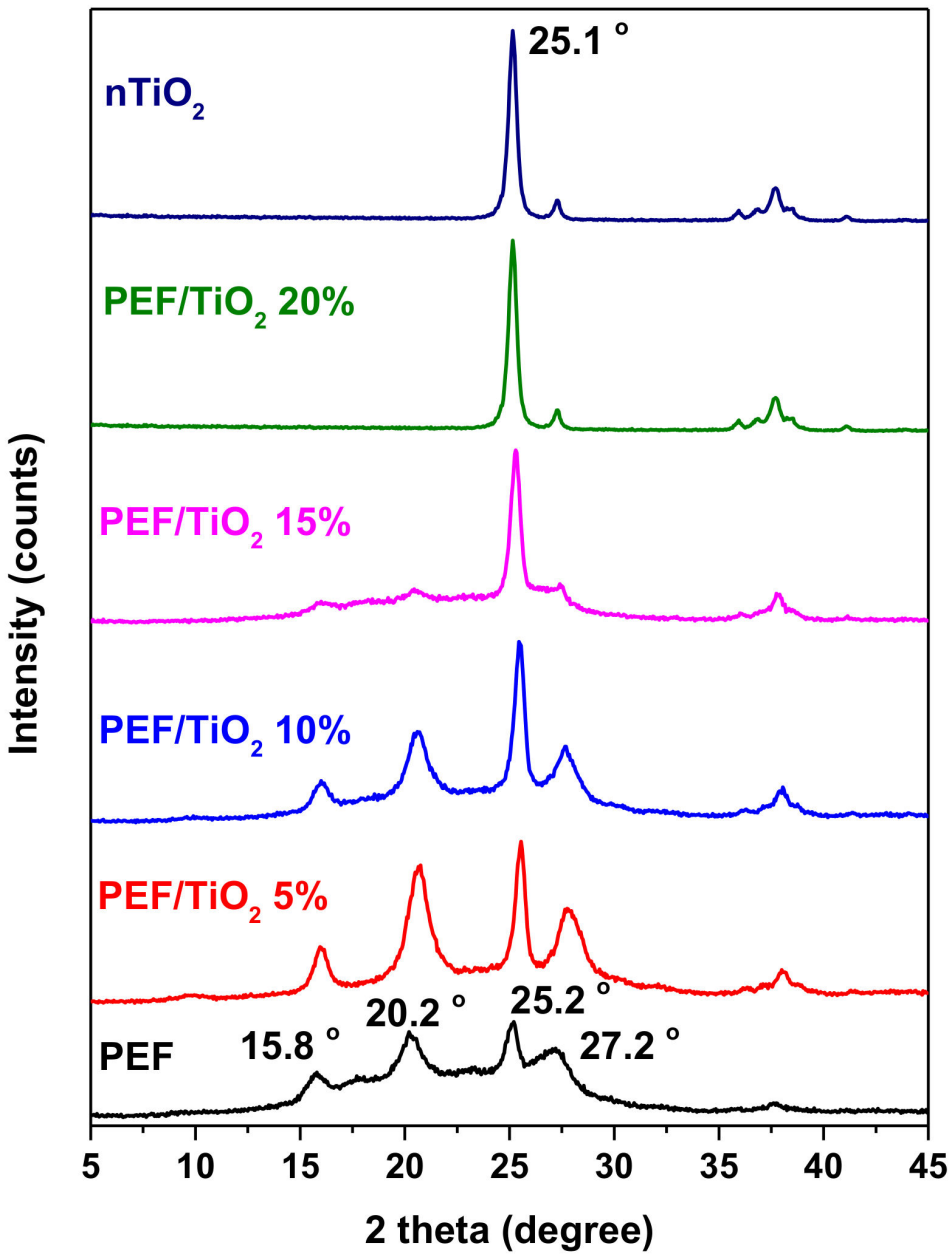
X-ray diffraction patterns of PEF and its nanocomposites with TiO_2_.

WAXD was employed in order to clarify the crystalline structure of PEF and TiO_2_ in the nanocomposites. The resulting diffraction patterns are shown in Figure 2. Neat PEF has some weak diffraction peaks at 15.8, 20.2, 25.2, and 27.2°, which reveal that it is crystallized in the β-crystal form [40]. Its crystal structure is sustained in the nanocomposites with 5 and 10% wt % TiO_2_, but is significantly reduced in concentrations of 15 and mainly 20 wt%, as evidenced by the very weak diffraction peaks. TiO_2_ powder exhibits an intense diffraction peak at 25.1°, which corresponds to the form of anatase. This peak is also present in the nanocomposites, indicating that the anatase structure remains the main form of TiO_2_ in the catalysts, which is the desired one since it is able to degrade compounds via photocatalysis [41]. Especially in the nanocomposites with 15 and 20 wt% TiO_2_, the nanofiller is well crystallized, with strong diffraction peaks, suggesting that it will retain its photocatalytic activity [32].

**Figure 3:**
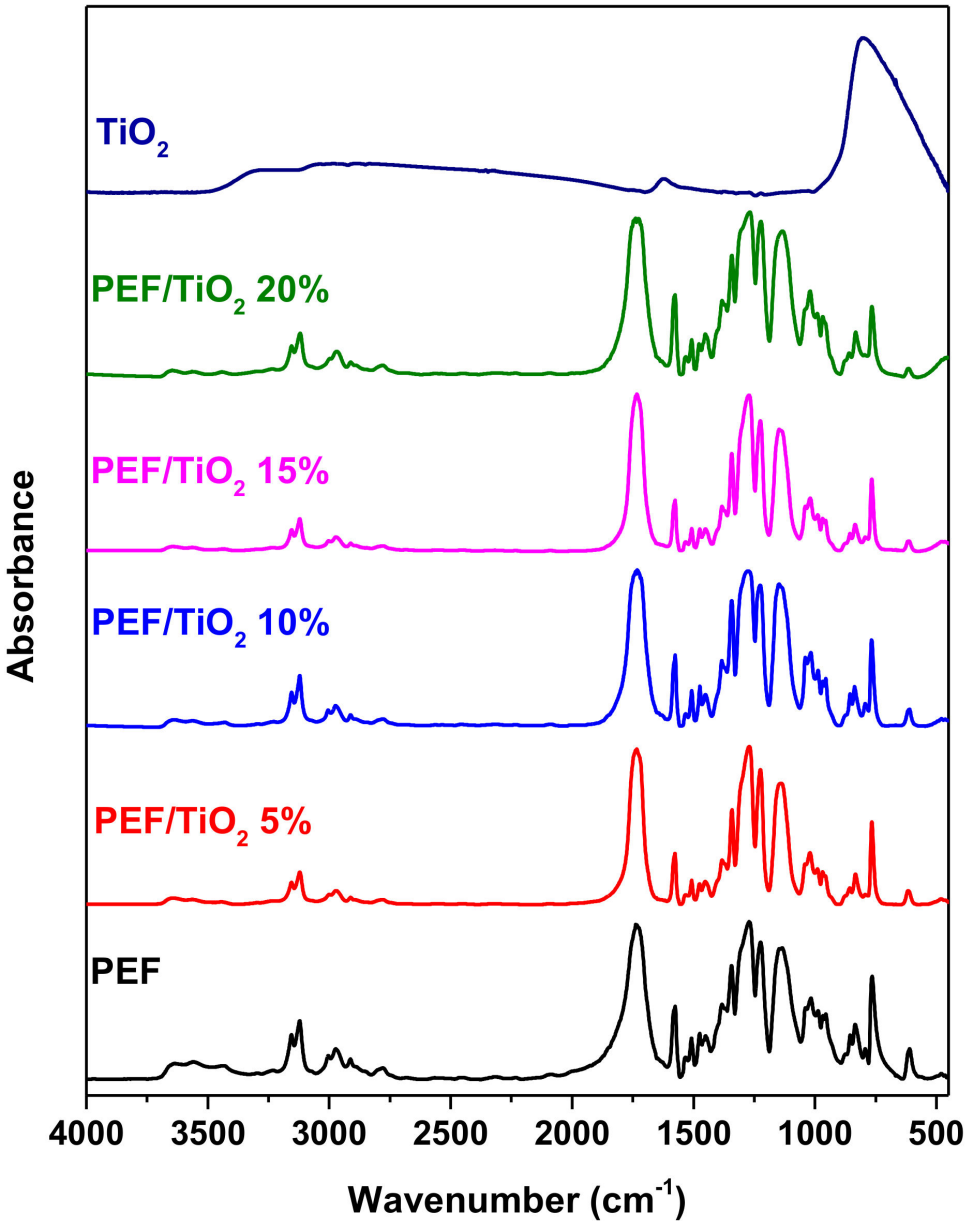
FTIR spectra of PEF and its nanocomposites with TiO_2_.

FTIR spectra of the polyesters were recorded to confirm their structure and possible interactions between polymer matrix and TiO_2_ (Figure 3). The spectrum of PEF exhibits absorption bands at 3645 cm^−1^ due to the RCH_2_OH hydroxyl group vibration, at 3563 cm^−1^ due to the O-H bending of the carboxylic hydroxyls, at 3435 cm^−1^ due to the O-H bending of the hydroxyl end groups of the polyester, at 3123 cm^−1^ and 3004 cm^−1^, due to the C-H bending of the furan ring, at 2976 cm^−1^ of the C-H bending vibrations, at 1737 cm^−1^ of the carbonyl group’s bending, at 1575 cm^−1^ due to the bending vibrations of the =C-H of the furan ring, at 1271 cm^−1^ and 1225 cm^−1^ due to the Csp^2^-O and Csp^3^-O bonds of the furan ring, and at 1136 cm^−1^ due to the C-O bending vibration of the ester group. The spectra of the nanocomposites do not exhibit noticeable differences compared with neat PEF, therefore the presence of TiO_2_ did not affect the chemical structure of the polymer. A small peak in the region 450–500 cm^−1^ that increases in area with increasing the nanofiller content is also observed and can be attributed to the Ti-O stretching vibration. Additionally, since the main peaks of PEF are recorded at the same positions in nanocomposites, it can be said that there are no covalent interactions or hydrogen bonding between PEF matrix and TiO_2_ nanoparticles.

**Figure 4:**
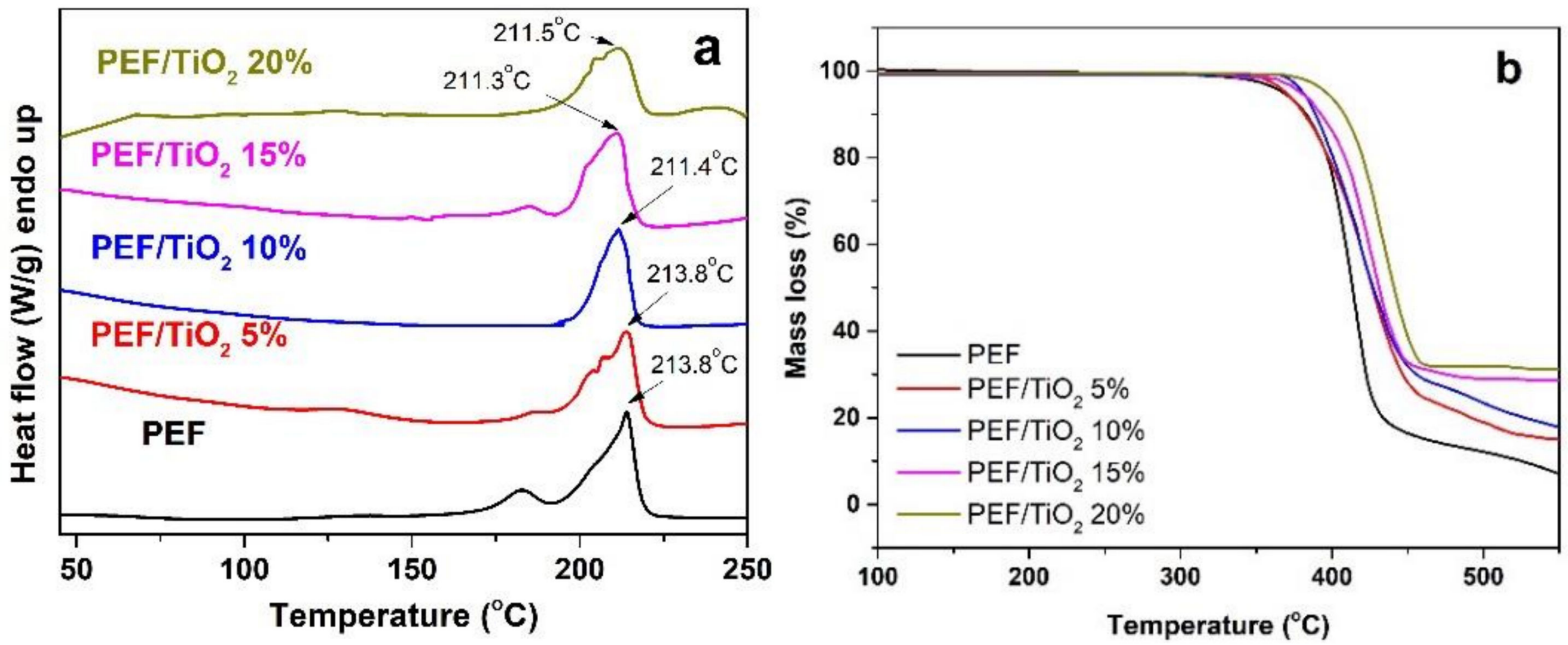
(**a**) DSC thermograms and (**b**) TGA thermograms of PEF and its nanocomposites with TiO_2_.

Thermal characterization of PEF and its nanocomposites was performed with DSC and TGA (Figure 4). The characteristic temperatures T_m_ and T_g_, as well as the crystallinity are presented in Table 1. The melting point of PEF appears at 213.8 °C, as a broad endothermic peak. After the incorporation of nTiO_2_, melting point values are not significantly affected in low filler content 5 wt%, but start to decrease slightly in the presence of 10–20 wt% nTiO_2_. This shift could be attributed to the lower degree of crystallinity that these nanocomposites have or due to the formations of imperfect crystals. As can be seen from enthalpy of fusion the degree of crystallinity reduces gradually by increasing TiO_2_ content. This is clear evidence that the nanoparticles, due to the higher amount added, restrict the ability of macromolecular chains to fold and form crystal structures. For this reason, the degree of crystallinity (X_c_) was reduced significantly in nanocomposites with 15 and 20 wt% TiO_2_, which is in good agreement with WAXD findings. In contrast, T_g_ is increased in the nanocomposites, indicating their hindering effect towards the movement of the macromolecular chains. 

The effect of TiO_2_ on the thermal stability of PEF was studied by TGA. The characteristic degradation temperatures and solid residue are also presented in Table 1. It can be seen that PEF is a thermally stable polyester, since it starts to degrade after 390 °C. Thermal stability is increased for all the nanocomposites, with increasing filler content, as evidenced by the T_max_. The amounts of solid residue at 550 °C are increasing, while increasing TiO_2_ content, as was expected, due to the high added TiO_2_ amounts. Furthermore, from these residues it is clear that the whole amount of added TiO_2_ was incorporated inside the polymer matrix.

### 2.2. Photocatalytic Degradation Process

#### 2.2.1. Effect of TiO_2_ Amount on Drug Removal

Due to the increased concern for the usage of immobilized TiO_2_ catalysts, all the synthetized catalysts (600 mg L^−1^ of each) with the concentration of TiO_2_ ranging from 5 to 20 wt%, were evaluated for the degradation of anti-inflammatory drugs mixture (5 mg L^−1^ of each compound), at inherent pH. 

The selected drugs are found very often in wastewaters as well as in surface waters. For example, IBU is a non-steroidal, antipyretic drug that is recognized with a huge global consumption rate [42] and has been detected in effluents of municipal WWTPs and in surface waters at concentrations levels between ng L^−1^ and µg L^−1^ [43]. ACM is a mild analgesic that is commonly used in combinatory drugs for the relief of fever, headaches, and some minor pains, which has been commonly detected in surface waters, ground waters, wastewaters, and even drinking waters, along with its toxic metabolites [44,45]. DCF is frequently detected in secondary effluent, surface water, and drinking water at concentrations range of from 1.2 to 4.7 μg L^−1^, respectively [46]. DCF is also one of the monitored pollutants by European Commission (EC), since itself and its transformation by-products pose a serious threat to public health and ecosystems according to the persistence and ecotoxicity in the water [47].

**Figure 5:**
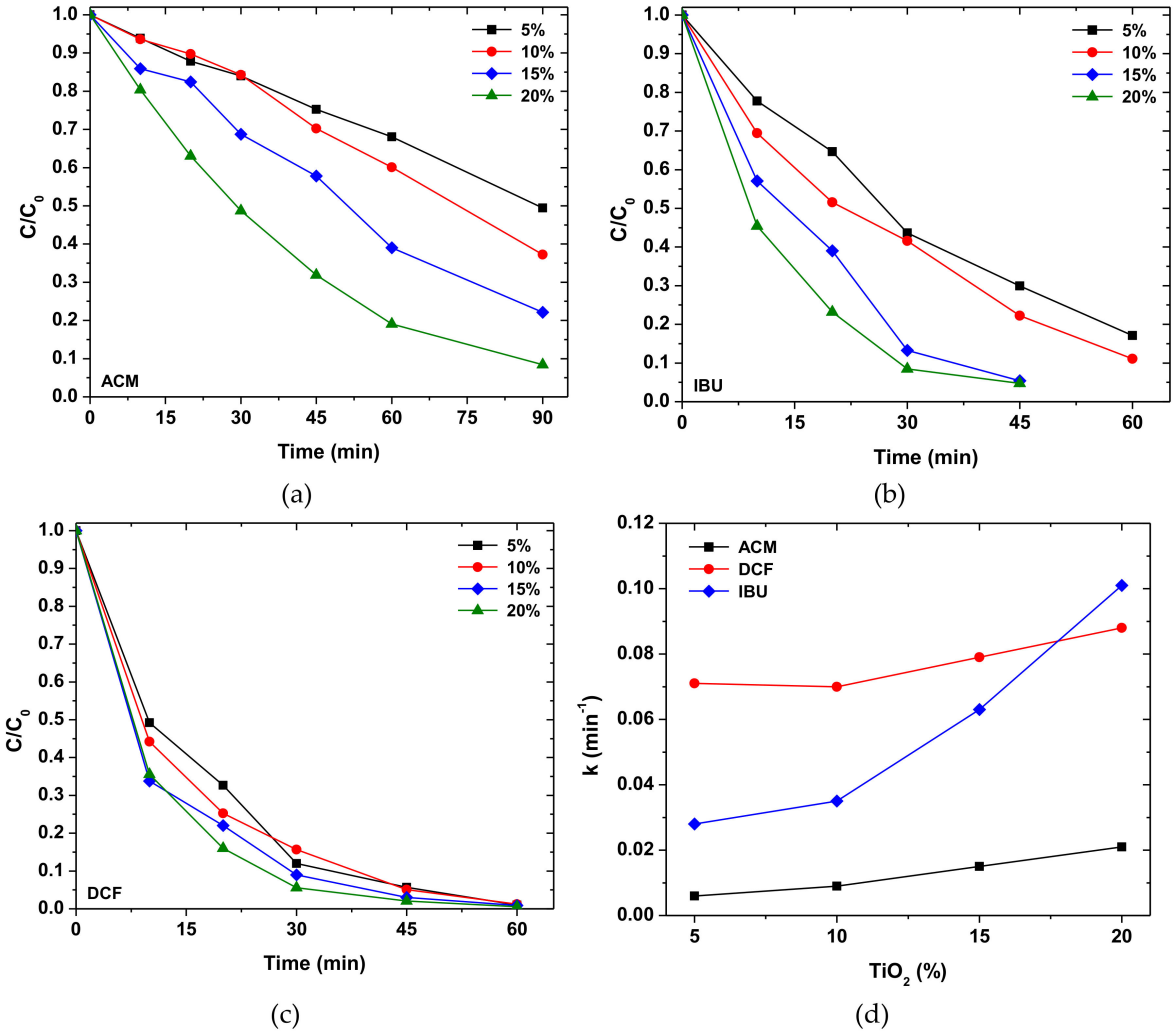
Effect of TiO_2_ amount immobilization at the efficiency of the anti-inflammatory drug mixture degradation under SSL, (**a**) ACM; (**b**) IBU; (**c**) DCF; (**d**) variation of degradation kinetic rate (k) versus TiO_2_ % content, (C_o_(drug) = 5 mg L^−1^); and (C(catalyst) = 600 mg L^−1^).

Mixture’s degradation by all studied catalysts, under SSL, follows first-order kinetics according to the high values of correlation coefficient, R^2^ = 0.99 (lnCo/C vs. time), for all the compounds. The kinetic profiles are depicted in Figure 5a–c, while the kinetic rate constants are in Figure 5d.

All four catalysts exhibit photocatalytic activity, while, as expected, the immobilized catalyst with the 20 wt% of TiO_2_ is the most efficient for the removal of the drug mixture, since the kinetics obtained are faster for all studied compounds. The elimination of all compounds by PEF/TiO_2_ 20 wt% catalyst was almost complete during the first 90 min of the procedure. Therefore, further experiments were only conducted for PEF/TiO_2_ 20 wt% catalyst. Preliminary adsorption experiments were also carried out using 5 mg L^−1^ of each compound as the initial concentration. Under dark conditions, negligible adsorption of the target compounds on the catalyst’s surface (600 mg L^−1^ of PEF/TiO_2_ 20 wt%) was observed.

#### 2.2.2. Effect of Drug Concentration 

**Figure 6:**
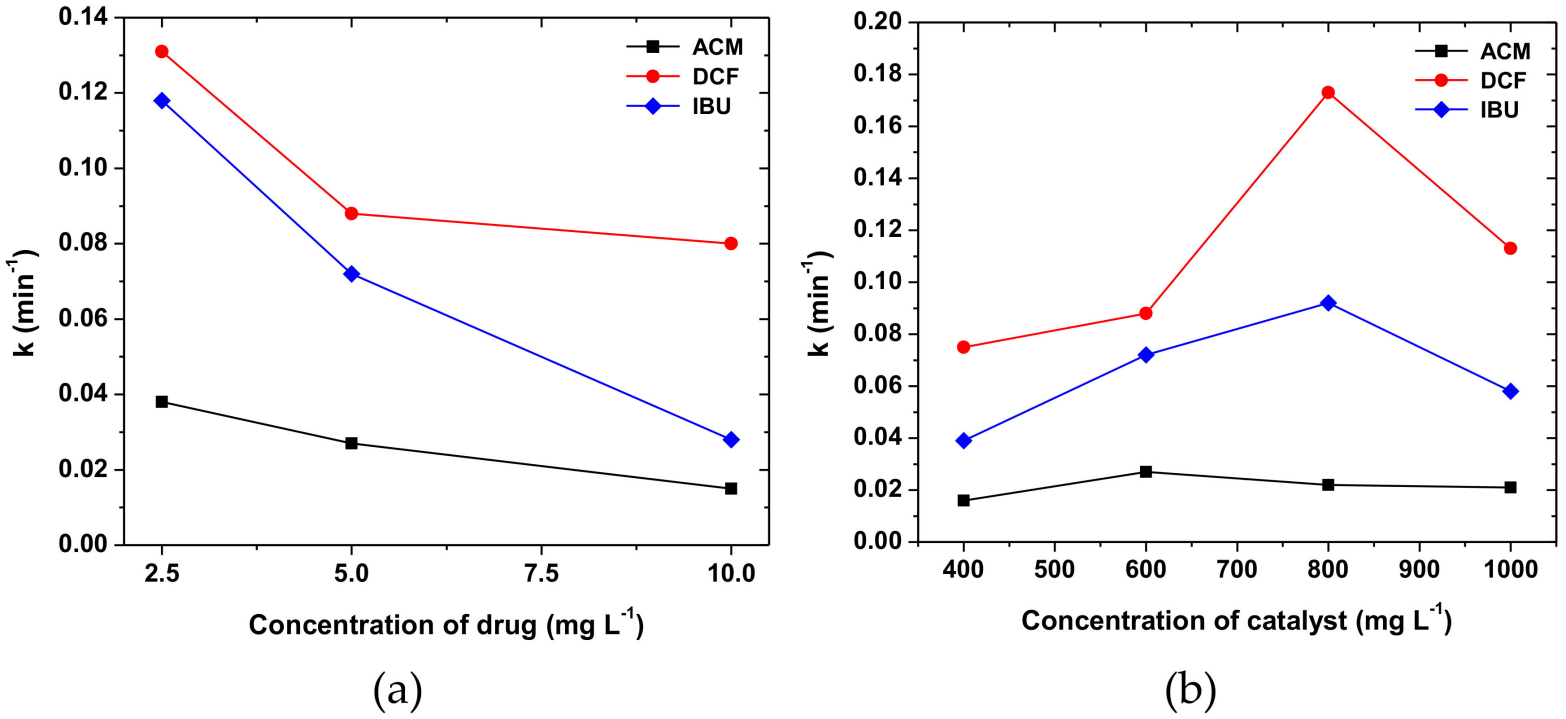

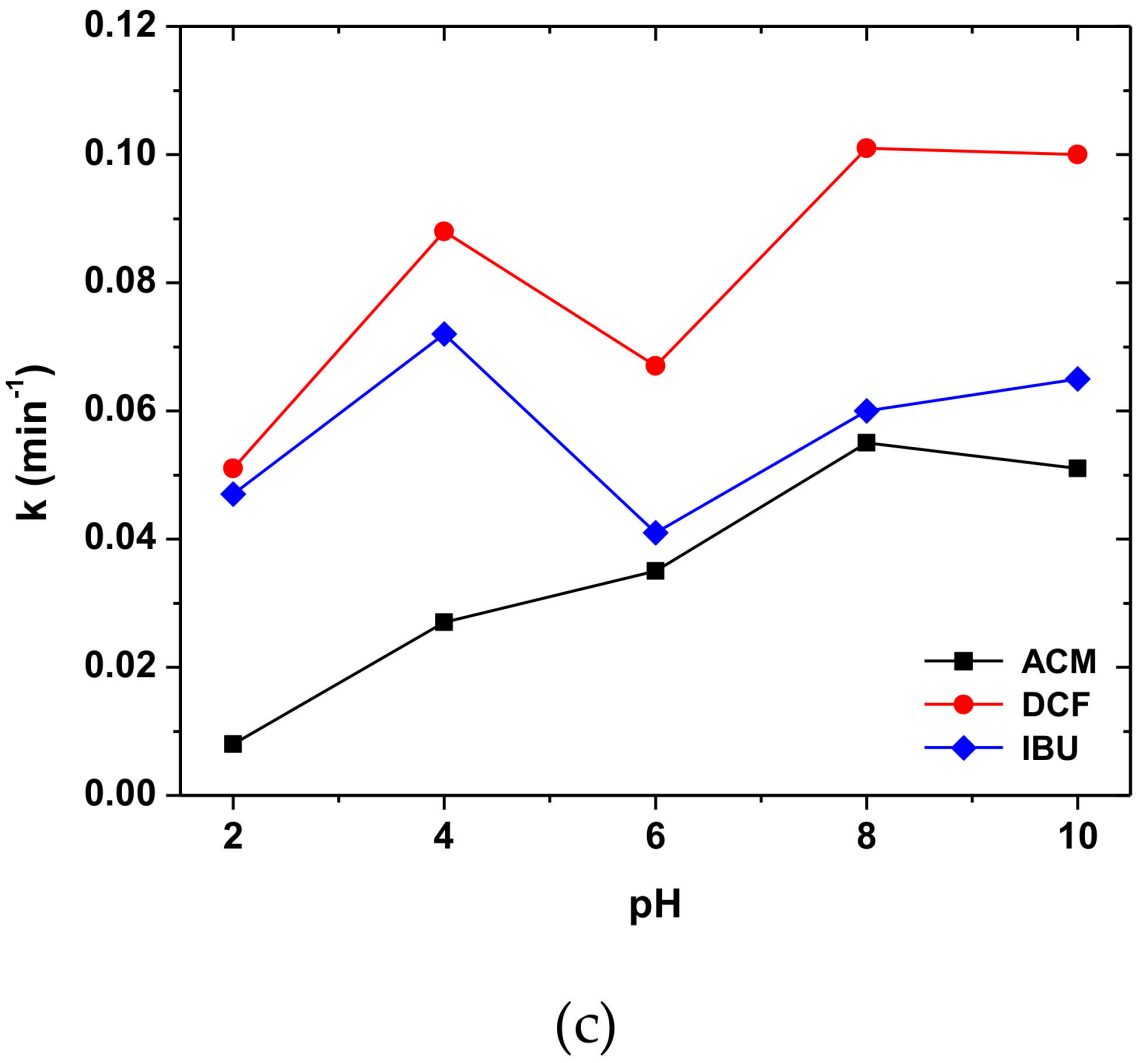
The effect of operational parameters on degradation kinetic rate (k) of anti-inflammatory drug mixture, (**a**) effect of mixture concentration (C(PEF/TiO_2_ 20 wt% = 600 mg L^−1^); (**b**) effect of catalyst loading (C_o_(drug) = 5 mg L^−1^); and (**c**) effect of pH, during photocatalytic treatment (C_o_(drug) = 5 mg L^−1^, C((PEF/TiO_2_ 20wt %) = 600 mg L^−1^).

Additionally, experiments were conducted in order to investigate the effect of the initial concentration of the mixture (2.5–10 mg L^−1^ of each compound), keeping the rest of the parameters constant. According to the results (Figure 6a), an increase in the concentration of the mixture leads to a decrease in the photocatalytic rate constants that could be attributed to the lower ratio of active oxidative species to the substrate molecules, which has also been reported elsewhere [48,49,50]. The concentration of 5 mg L^−1^ for each drug was selected for further experiments and the effect of several parameters to pollutants removal was evaluated, since this concentration enables the proper monitoring of the degradation kinetics. The degradation curves obtained for each drug are given in detail in the Supplementary Materials (Figure S1).

#### 2.2.3. Effect of Catalyst Loading

Different concentrations of PEF/TiO_2_ 20% catalyst, ranging from 400 to 1000 mg L^−1^, were employed, in order to study the effect of catalyst’s concentration on the degradation of the mixture (Figure 6b). The detailed degradation curves are given in the Supplementary Materials (Figure S2). Generally, when increasing the concentration of the catalyst, the surface of the particles also increases, making available a higher number of active sites for adsorption and generation of the oxidant species, until an optimum catalyst mass corresponding to the maximum absorption of photons reaching the catalytic bed. Figure 6b represents the results of the above study. Obviously, until 800 mg L^−1^, as the concentration rises, there is an increase at the rate constant of each compound. Above the concentration until 800 mg L^−1^, the rate constant either reaches a plateau (for ACM) or decreases (for IBU and DCF). 

#### 2.2.4. Effect of pH

During the heterogeneous photocatalytic treatment, the pH of the semiconductor’s solution can be a determining factor for the efficiency of the whole procedure. The pH value affects the charge of the catalyst’s surface, as well as the reactant’s charge and cause either electrostatic repulsion or attraction between them [51,52]. In our case, TiO_2_ is amphoteric and consequently and its point of zero charge (pzc) is widely reported at pH ∼6.7. Therefore the TiO_2_ surface is positively charged in acidic media (pH < 6.7), whereas it is negatively charged under alkaline conditions (pH > 6.7) [53]. Additionally, the PEF polymeric immobilization of TiO_2_ may be charged as either negative or positive, since its furanic ring or carboxyl groups are pH dependent. For example, the carboxylic acid groups are feasibly ionized to release the protons under basic condition and the carboxylate anion and the proton tend to combine under acidic condition [54].

As presented at Figure 6c, the effect of pH on the photocatalytic degradation of the anti-inflammatory drug mixture was studied for pH values ranging from two to 10. The effect of pH for each drug is also given at Supplementary Materials (Figure S3), expressed as degradation curves. For very acidic conditions (pH 2) the degradation kinetics are obviously slow for all the studied compounds. This is probably attributed to the combined effect of the protonation process and the H^+^ excess in the solution, which does not favor the generation of hydroxyl radicals [55]. For ACM the degradation kinetic increases by the increase of pH until pH 8. For very basic pH, there is a small decrease. The pKa of ACM is 9.71 [56], which means that it is presented in cationic form for pH < 9.71, therefore, when the catalyst is negatively charged (pH > 6.7), there is the maximum adsorption of ACM to the catalyst’s surface, which is pH 8 in our study. In the case of IBU, the higher reaction rate was observed for acidic pH (pH = 4), which is consistent with findings that acidic conditions favor IBU degradation [57]. IBU is a weakly acidic compound with pKa = 4.8 [58], so at pH values that exceeded its pKa, the molecule was present in its an-ionic form, which decreases the efficiency of the photocatalytic treatment.

Finally, for DCF, there were no significant differences as far as the degradation kinetic is concerned for pH, 4.8 and 10. The pKa of DCF is approximately 4.50 [59], so at pH 6, which is close to pzc of TiO_2_, it is probably negatively charged that does not favor the adsorption on the catalyst’s surface. The increase at pH 8 and 10, compared to pH 6, for IBU and DCF can be explained by the increase in generation of hydroxyl radicals, which is favoured at basic conditions [55]. For further experiments, the inherited pH (approximately pH = 4) was selected.

#### 2.2.5. Mineralization Process

**Figure 7:**
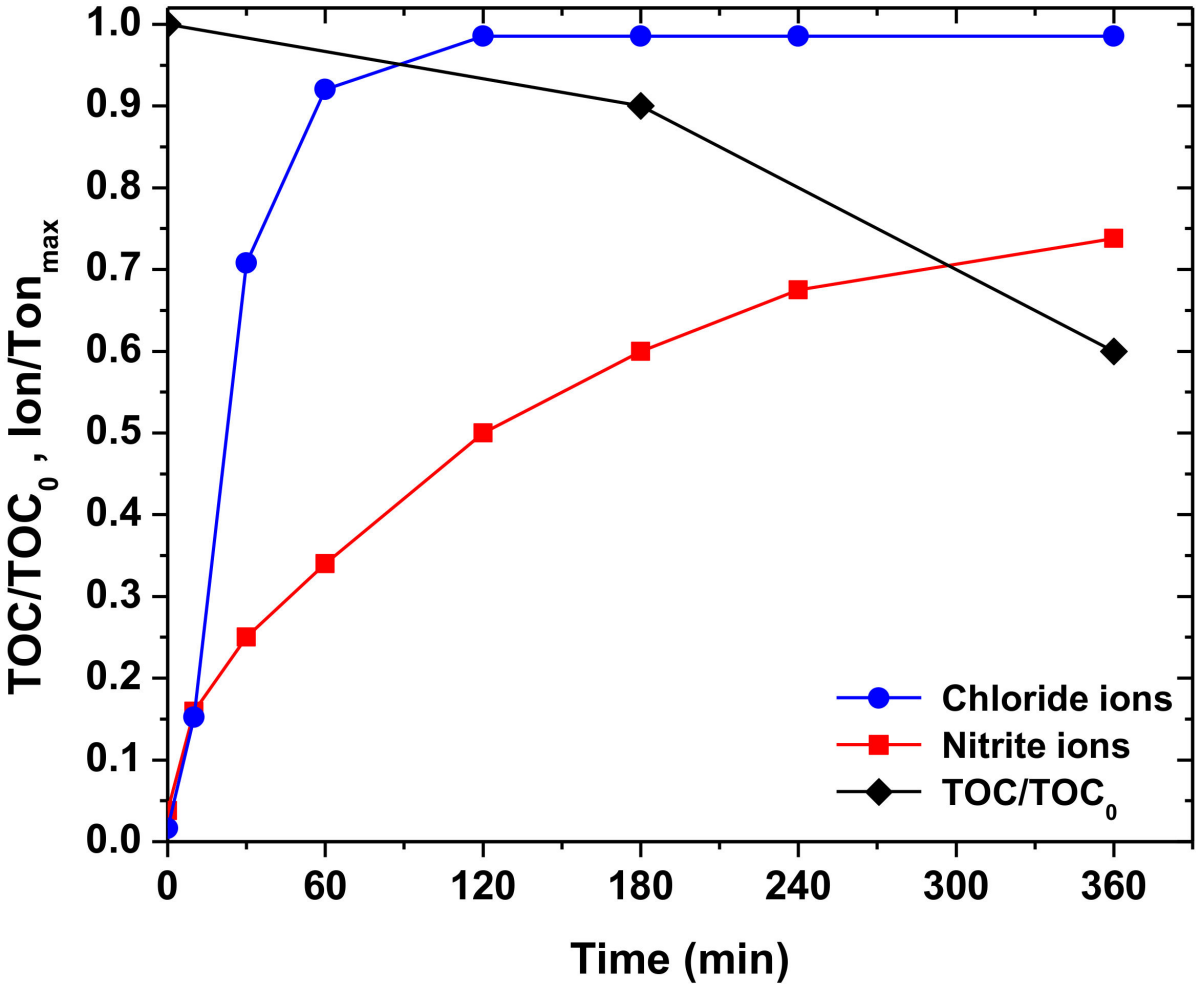
The effect of operational parameters on degradation kinetic rate (k) of anti-inflammatory drug mixture, (**a**) effect of mixture concentration (C(PEF/TiO_2_ 20 wt% = 600 mg L^−1^); (**b**) effect of catalyst loading (C_o_(drug) = 5 mg L^−1^); and (**c**) effect of pH, during photocatalytic treatment (C_o_(drug) = 5 mg L^−1^, C((PEF/TiO_2_ 20 wt%) = 600 mg L^−1^).

In order to assess the efficiency of PEF/TiO_2_ 20% for the degradation of the anti-inflammatory drug mixture, mineralization studies were conducted. The concentration of each drug was 5 mg L^−1^, while the concentration of the catalyst was 600 mg L^−1^. The mineralization was studied for 360 min of irradiation (Figure 7). Although, all compounds degrade quite fast (during the first 90 min of treatment), TOC removal follows a slow rate reaching approximately 50% decrease after 360 min, demonstrating that the photocatalytic treatment needs prolonged irradiation times for leading to complete mineralization. This is probably due to the transformation products formed from the degradation of parent compounds. The chloride ions released from the degradation of DCF, reaches almost 100% from the first 120 min of treatment, probably indicating that the transformation products released are chloride-free or that the transformation products of DCF also degrade quickly. Nitrite ions were also released during the photocatalytic treatment, from the degradation of DCF and ACM. Their release reached approximately 70% at the 240 min of irradiation, while no nitrate ions are observed.

#### 2.2.6. By-Product Evaluation

**Figure 8:**
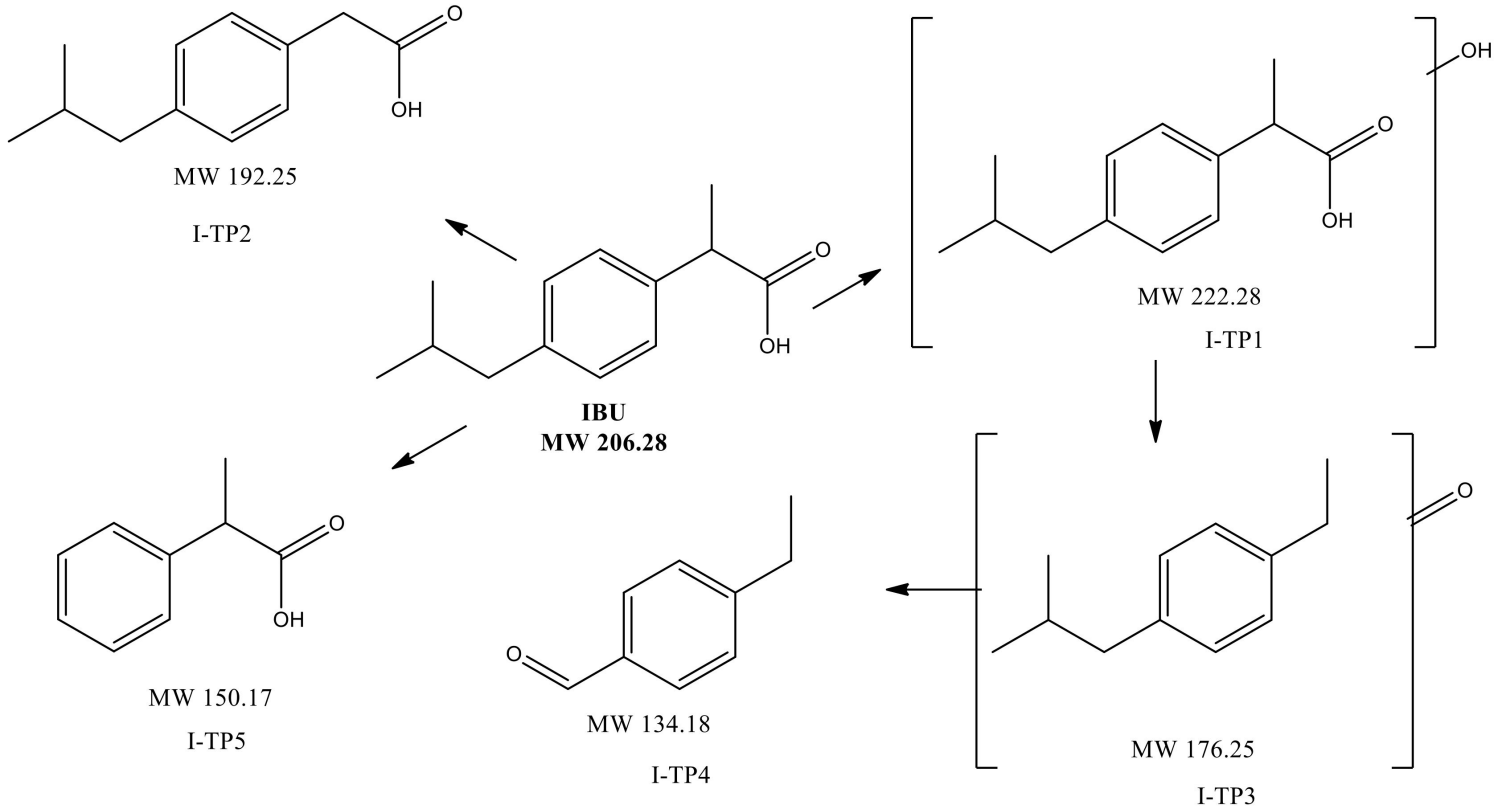
TPs products generated from IBU by PEF/TiO_2_ 20 wt% under SSL.

An attempt to identify the TPs generated by the heterogeneous photocatalytic degradation of the anti-inflammatory drug mixture by PEF/TiO_2_ 20 wt% under SSL was also conducted. The data obtained by high-resolution mass spectra, are given by Table 2. In this study, five TPs generated during IBU photodegradation were identified (Figure 8).

According to the generated TPs, IBU mostly goes under degradation by hydroxylation, demethylation, decarboxylation, cleavage of isobutyl moiety, and oxidation of hydroxyl groups. The TPs generated from the degradation of IBU are in accordance to those obtained in previous studies by the oxidation techniques [60,61,62,63,64,65].

**Figure 9:**
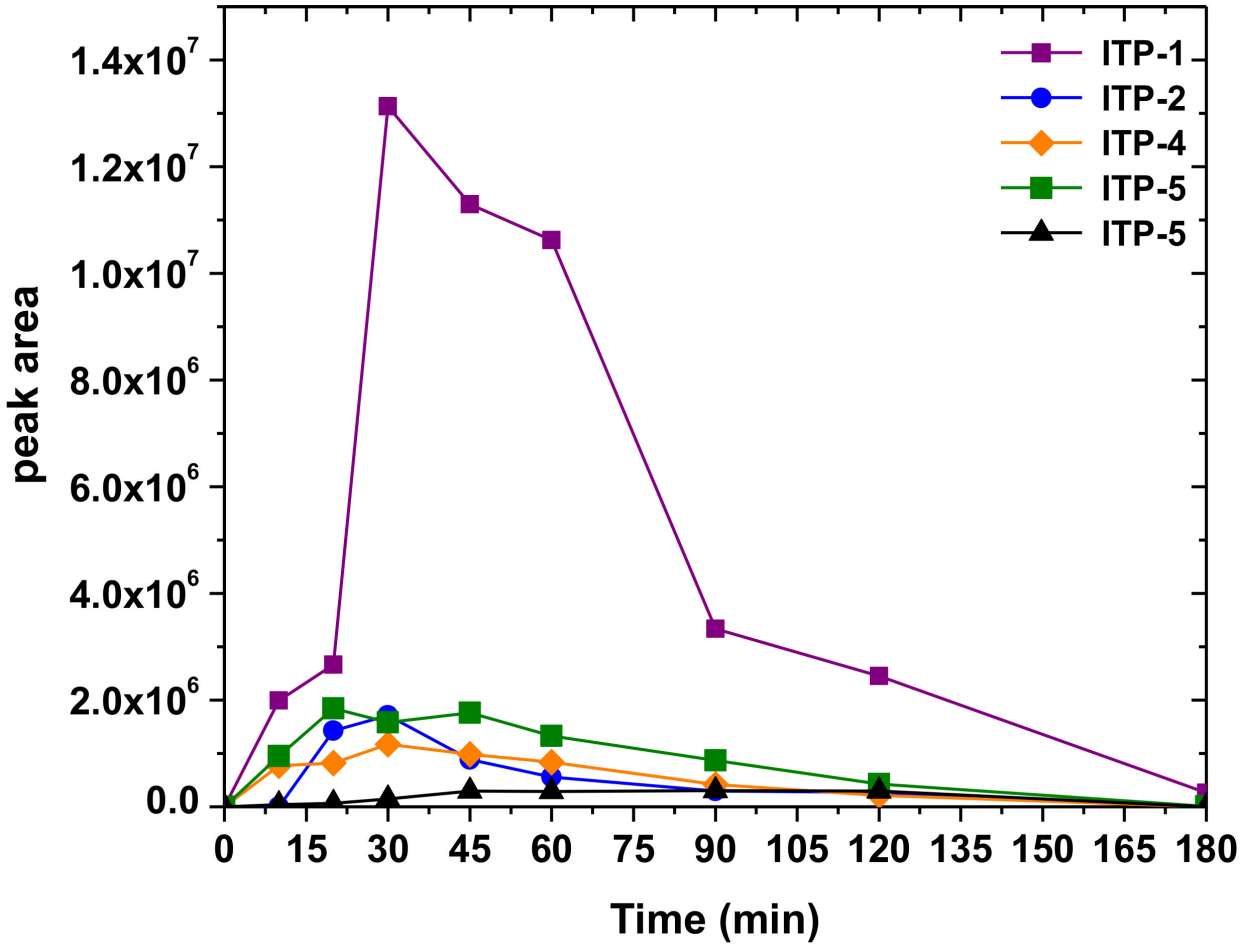
Evolution profiles of TPs generated from IBU by PEF/TiO_2_ 20 wt% under SSL, C_0_(drug) = 5 mg L^−1^, and C(PEF/TiO_2_ 20 wt%) = 600 mg L^−1^.

Initially, HO• attack IBP molecule to form mono-hydroxylated species (I-TP1). The above TP, induced decarboxylation and formation of I-TP3, which was further transformed to I-TP4. Additionally, a methyl group of IBP molecule was attacked by HO• to form a demethylated intermediate (I-TP2). In another third pathway, detachment of isobutyl group occurs, which leads to the formation of 2-phenylpropanoic acid (I-TP5). The evolution profiles of the above TPs are given in Figure 9. TPs emerged within the first 15 min of irradiation and remained abundant in the solution for 120 min of the applied.

**Figure 10:**
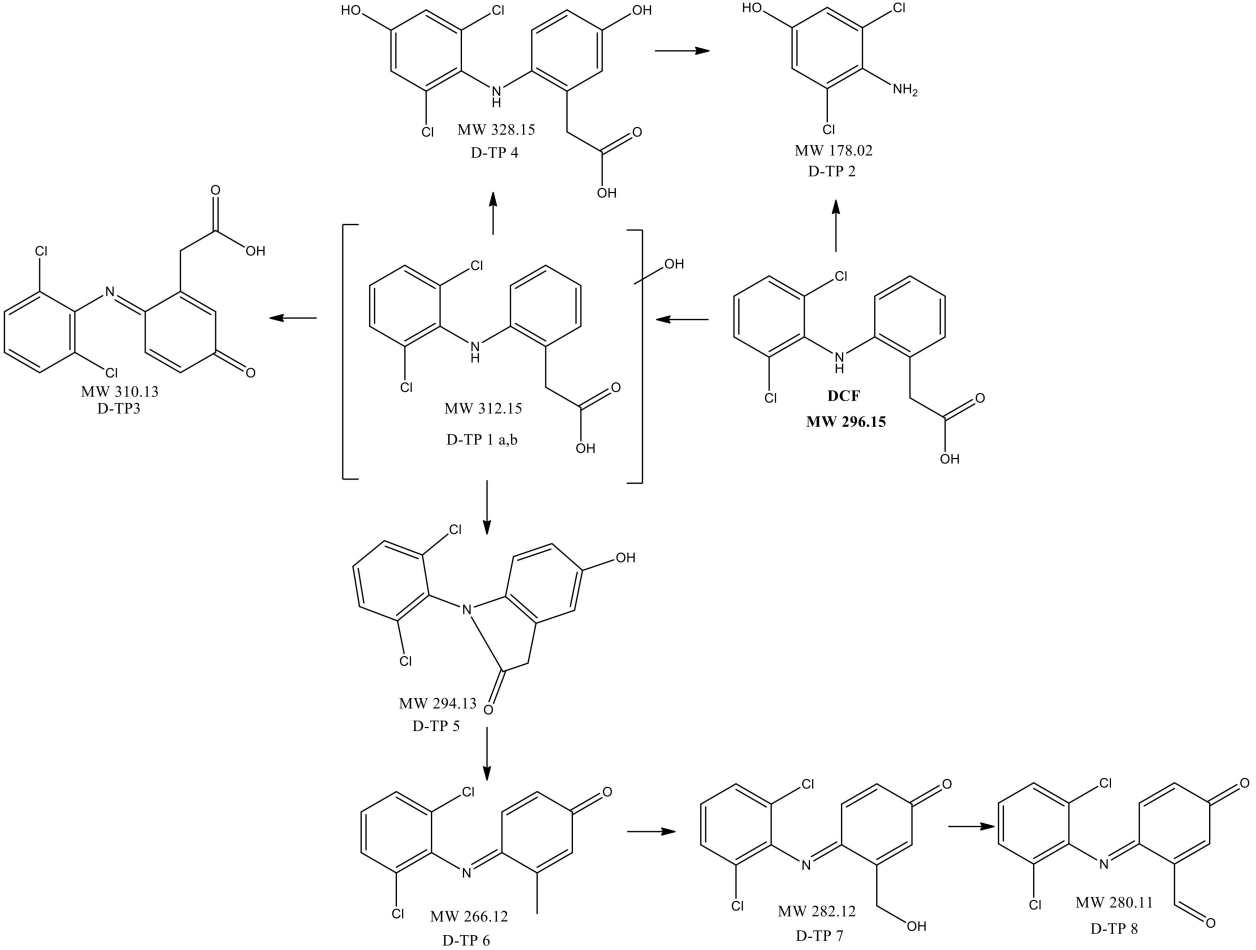
TPs products generated from DCF by PEF/TiO_2_ 20 wt% under SSL.

**Figure 11:**
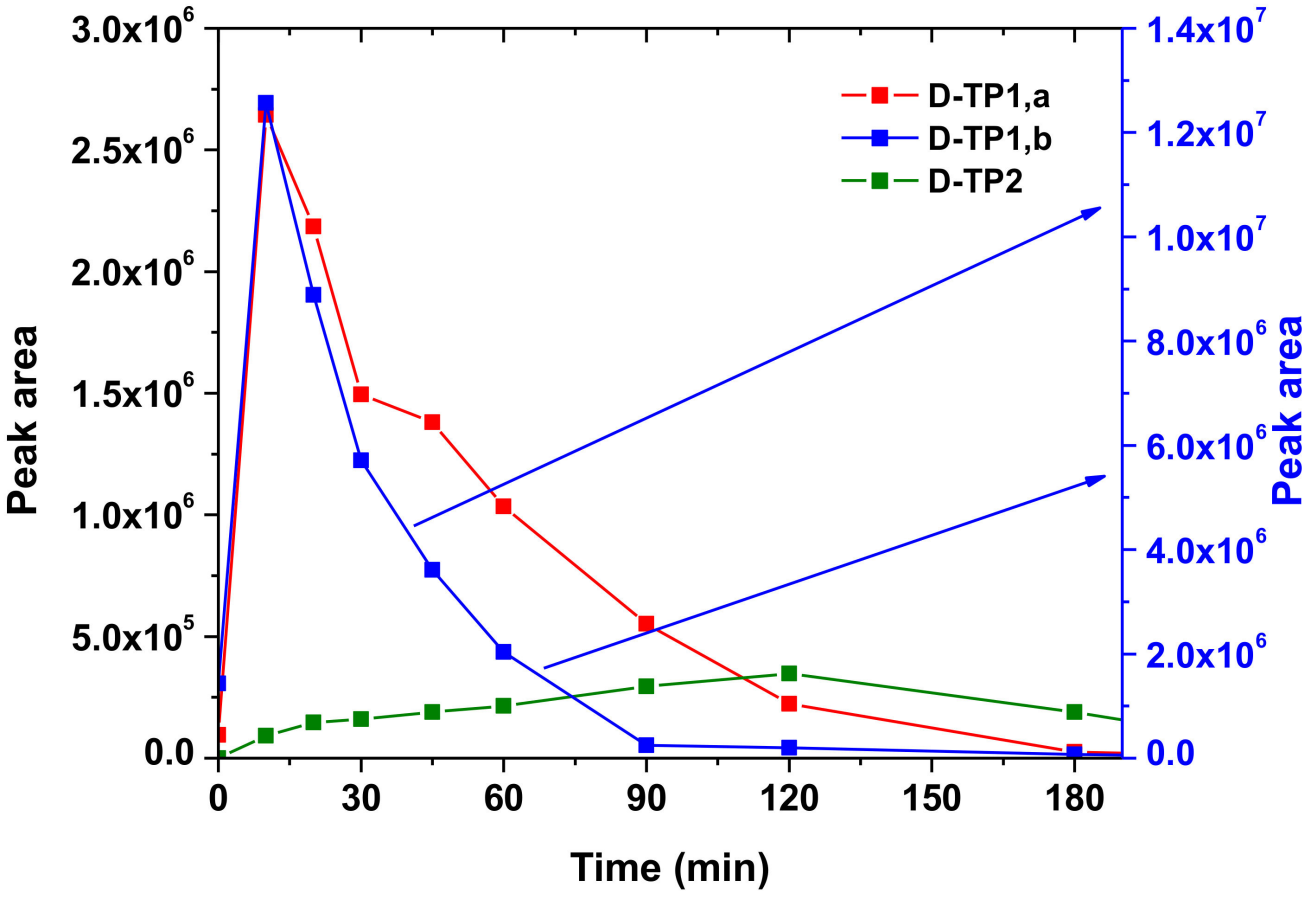
Evolution profiles of TPs generated from DCF by PEF/TiO_2_ 20 wt% under SSL, C_0_(drug) = 5 mg L^−1^, and C(PEF/TiO_2_ 20 wt%) = 600 mg L^−1^.

In the case of DCF, nine TPs were identified (Figure 10). DCF transformation under oxidizing experimental conditions appears to proceed mainly by oxidation and hydroxylation reactions between chloroaniline and phenylacetic acid. The reaction of HO• with the aromatic ring forms a resonance-stabilized carbon-centered radical with the subsequent elimination of hydrogen radical, leading to the formation of the mono hydroxylated species (D-TP1,a,b). These mono hydroxylated TPs were further oxidized, resulting in the production of D-TP4 and D-TP5, while D-TP6 and their decarboxylated derivatives were formed by the loss of their carboxylic moiety. TP6 was probably further transformed to D-TP7 and D-TP8. Furthermore, the presence of D-TP3 suggests that one (of the four) monohydroxylated derivatives is further oxidized into the correspondent keto-derivative. Additionally, the formation of D-TP2 indicates the cleavage of the C–N bond of DCF as a preferential route, which originated from a series of C–N cleavage products. D-TP1,a,b and D-TP2 were mainly detected, at higher relative intensities compared to the other TPs, so only their evolution profiles were obtained, depicted in Figure 11. All are detected by the first 10 min of the process. D-TP2 has a maximum at 120 min. D-TP1,a,b decrease after the first 10 min, reaching undetectable levels at 180 min. The proposed degradation pathway has also been indicated by several studies regarding DCF oxidation [66,67].

**Figure 12:**
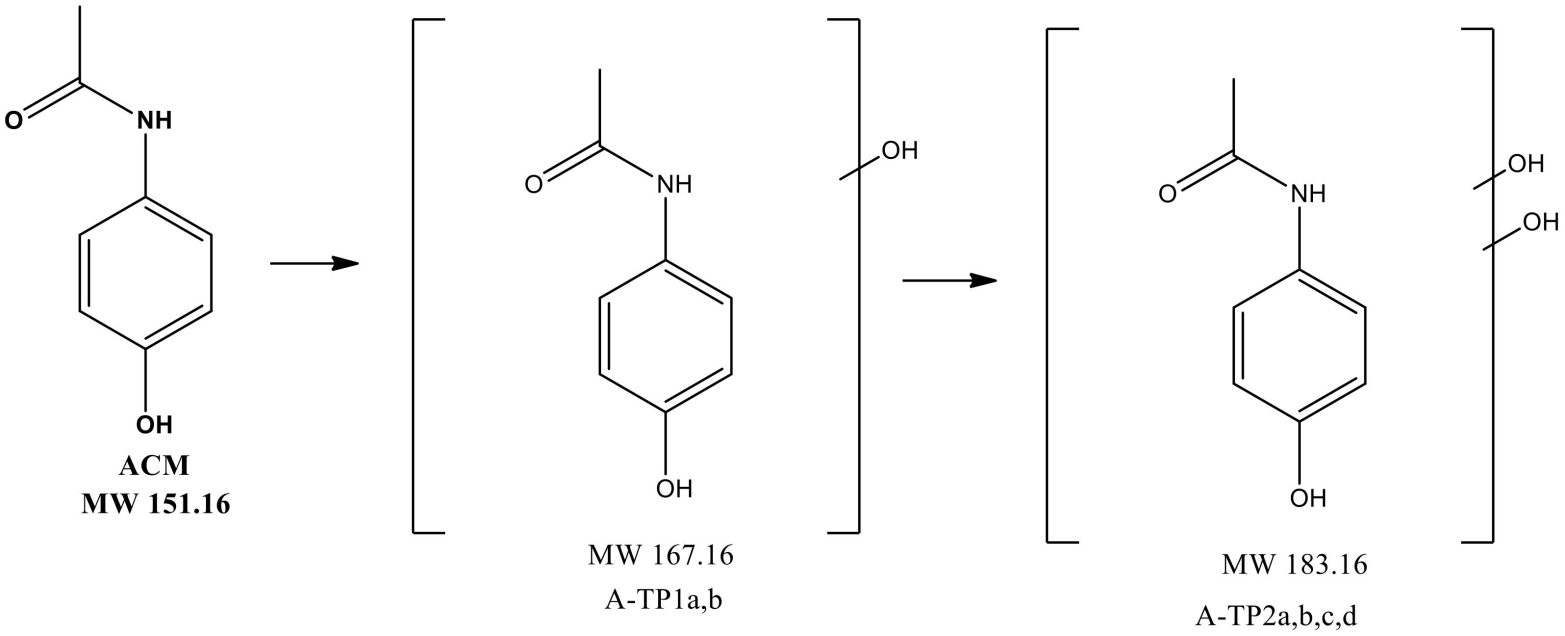
TPs products generated from ACM by PEF/TiO_2_ 20 wt% under SSL.

In this study, hydroxylation can be considered as the main pathway of ACM transformation, since both the mono and di-hydroxylated derivatives of ACM have been identified (A-TP1,a,d and A-TP2,a,b,c,d) (Figure 12). The oxidation and subsequent degradation of ACM via hydroxylation is also proposed by several studies over ACM degradation [68,69].

**Figure 13:**
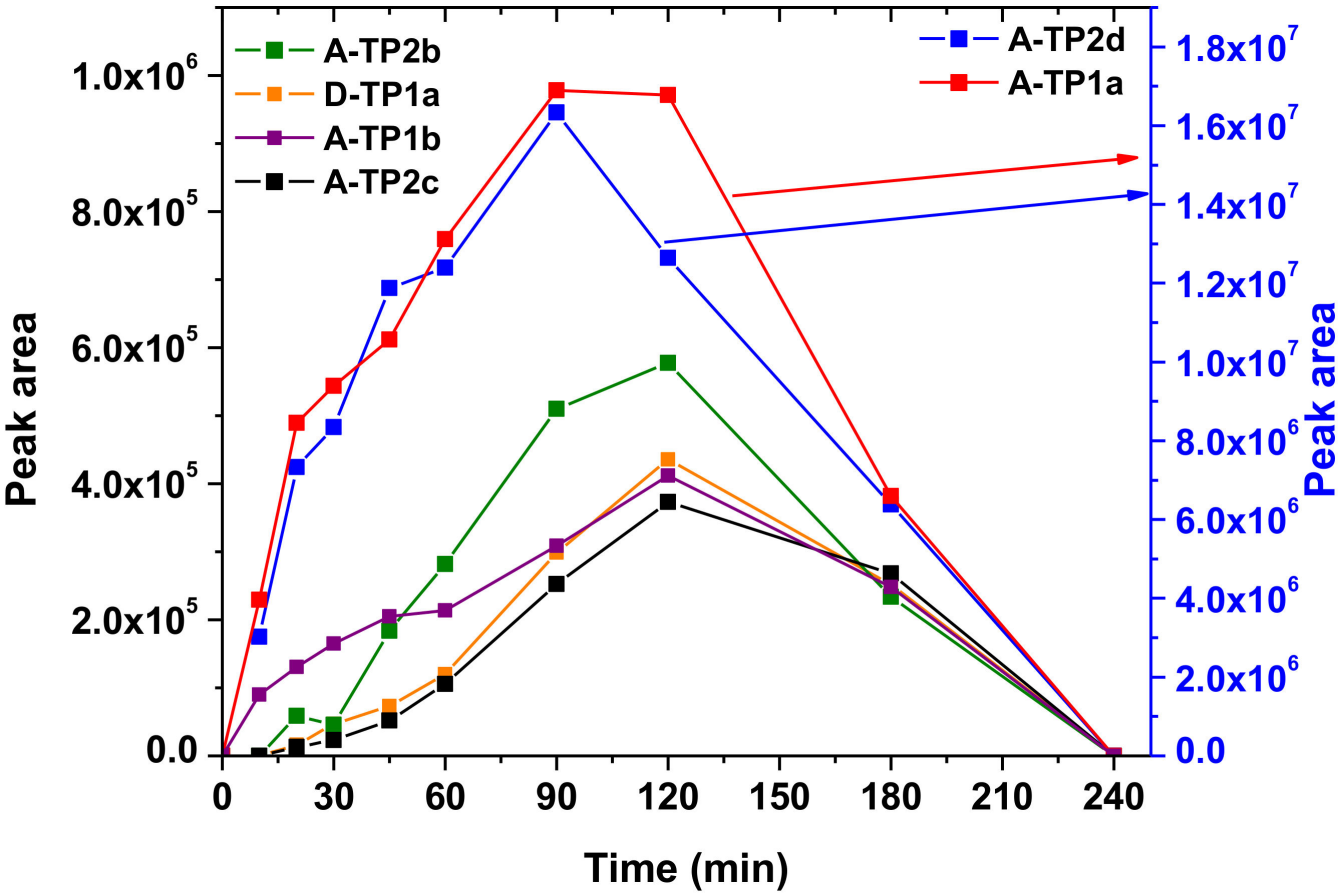
Evolution profiles of TPs generated from ACM by PEF/TiO_2_ 20 wt% under SSL.

The generated TPs present a peak at 120 min and are eliminated completely at 240 min of the photocatalytic procedure, as indicated in Figure 13.

All in all, the generated HO• from PEF/TiO_2_ 20% TiO_2_ under SSL, appear to be the main reactive specie to the photocatalytic degradation of all the three studied compounds in the anti-inflammatory drug mixture.

#### 2.2.7. Reusability Studies

From practical point of view, the stability of photocatalysts is an essential qualification feature. For this reason, the reusability of PEF/TiO_2_ 20% wt% was studied in three utilization cycles under simulated solar irradiation. The experimental procedure was similar to that described in Section 3.5, but in this case, after each reaction, the material was rinsed with water and dried in an oven at 50 °C for 8 h before being reused in two additional consecutive photocatalytic experiments. It can be found that there is no significant change in the rate constants of the three target drugs in the three consecutive experiments. According to the results a reduction in the rate constant between 12% and 18% was observed in the third run with respect to the second run for all the studied compounds, indicating the good stability of these materials under the applied reaction conditions, which is attributed to the enhanced binding nature of PEF towards TiO_2_.

## 3. Materials and Methods

### 3.1. Materials

2,5-furan dicarboxylic acid (purum 97%), ethylene glycol anhydrous 99.8% (EG), tetrabutyltitanate (TBT) catalyst of analytical grade, Ibuprofen (IBU), acetaminophen (ACM) and diclofenac (DCF) sodium were obtained from Sigma-Aldrich (Steinheim, Germany). Titanium dioxide P25 from Evonik (Marl, Germany) (particle size 20–30 nm; crystal structure: ~80% anatase and 20% rutile; surface area: 56 m^2^/g, zero point of charge ~6.3–6.8) was used as photocatalyst. LC-MS-grade methanol was supplied by Merck (Athens, Greece). Ultrapure water for the experiments was obtained from a Millipore Waters Milli-Q water purification system (Merck SA Hellas, Athens, Greece).

### 3.2. Polyester Synthesis

PEF was synthesized through the two-stage melt polycondensation (esterification and polycondensation) in a glass batch reactor, as described in our previous work [70]. 2,5-dimethylfuran-dicarboxylate (DMFD) was prepared using 2,5-FDCA, anhydrous methanol and concentrated sulfuric acid, as described previously [70]. DMFD and ethylene glycol in a molar ratio of diester/diol = 1/2.2 were charged into the reaction tube of the polyesterification apparatus with 400 ppm of TBT. The reaction mixture was heated at 150 °C under argon flow for 2 h and stirring speed 350 rpm, at 160 °C for an additional 2 h and finally at 170 °C for 1 h. This first step (transesterification) is considered complete after the collection of almost all the theoretical amount of CH_3_OH, which was removed from the reaction mixture by distillation and collected in a graduate cylinder. In the second step of polycondensation, vacuum (5.0 Pa) was applied slowly over a time of about 30 min to remove the excess of diol, to avoid excessive foaming and to minimize oligomer sublimation, which is a potential problem during the melt polycondensation. The temperature was gradually increased (1 h) to 220 °C, while stirring speed was also increased to 720 rpm. The reaction continued at this temperature for 2 h. Subsequently, the temperature was increased to 230 °C for 2 h and to 240 °C for an additional 1 h. After the polycondensation reaction was completed, the polyesters were removed from the reactor, milled, and washed with dissolution in TFA/chloroform 1/4 v/v and precipitation in cold methanol.

### 3.3. Photocatalyst Preparation

PEF nanocomposites with TiO_2_ were prepared by the solution mixing method, with a mixture of dicloromethane/chloroform/trifluoroacetic acid as a mutual solvent. The desired amount of nanofiller was first added into the solvent at a concentration of 1 mg/mL, and the mixture was subjected to sonication for 1 h to obtain a uniform dispersion. At the same time, PEF was completely dissolved in the solvent mixture at a concentration of 20 mg/mL. The PEF solution was then mixed with the nanofiller suspension followed by stirring and sonication for 30 min. The solvent was allowed to evaporate in air for three days with slight stirring for the first day and then at 50 °C for two days under a vacuum. After complete solvent removal, the nanocomposites were obtained in the form of powder. Powder was melt pressed in a hot press at 220 °C for 10 min in order to obtain films with 1 mm thickness. According to this procedure PEF/nTiO_2_ nanocomposite films containing 5, 10, 15, and 20 wt% TiO_2_ were obtained.

### 3.4. Photocatalyst Characterization

Number-average molecular weight (M_n_) of PEF was measured by Gel permeation chromatography (GPC) using a Waters 150 °C apparatus (Milford, MA, USA) equipped with differential refractometer as a detector and three ultrastyragel (103, 104, 105 Å) columns in series. Hexafluoroisopropanol was used as mobile phase at a flow rate 0.5 mL/min at 40 °C. Calibration was performed using polystyrene standards with a narrow molecular weight distribution.

Fourier transform infrared spectroscopy (FTIR) spectra of all the samples were obtained using a Perkin-Elmer FTIR spectrometer (Dresden, Germany), model Spectrum One, with the use of KBr discs. The IR spectra were obtained in absorbance mode and in the spectral region of 400–4000 cm^−1^ using a resolution of 4 cm^−1^ and 64 co-added scans.

Wide-Angle X-ray Diffraction (WAXD) patterns of the samples were recorded using a MiniFlex II XRD system from Rigaku Co (Chalgrove, Oxford, UK), with CuK_α_ radiation (λ = 0.154 nm) in the angle 2θ range from 5 to 60 degrees.

For thermal analysis measurements a Perkin-Elmer (Dresden, Germany), Pyris Diamond differential scanning calorimetry (DSC), coupled with an Intracooler 2P cooling accessory, was used. Samples of 10 ± 0.1 mg sealed in aluminium pans were used to test the thermal behavior of the polyesters. The samples were heated from 30 °C to 300 °C in a 20 mL/min flow of N_2_ with heating rate 20 °C/min in order to observe the melting temperature of the as received polyesters. The samples first were held at that temperature for 2 min, then quenched and rescanned again until 300 °C.

Thermogravimetric analysis (TGA) measurements were carried out by a STA 449C (Netzsch-Gerätebau, GmbH, Selb, Germany) thermal analyzer from room temperature up to 600 °C with 20 °C/min heating rate and 30 mL/min flow of N_2_ (99.9%).

Scanning electron microscopy (SEM) was carried out using a JEOL JMS-840A scanning microscope (Tubney WoodsAbingdon, Oxfordshire, UK) was equipped with an energy-dispersive X-ray (EDX) Oxford ISIS 300 micro-analytical system.

### 3.5. Photocatalytic Degradation Experiments

Photocatalytic experiments under UV-Vis irradiation were carried out in a solar simulator Atlas Suntest CPS+ (Linsengericht, Germany). Illumination was provided with a xenon lamp (1.5 kW) at an irradiance of 700 W m^−2^. Irradiation experiments were performed using a Pyrex glass reactor containing 100 mL of aqueous solutions, at various amounts of catalyst (400–1000 mg L^−1^), while the pH ranged from two to 10. The effect of pH was studied by adjusting the solutions with HCl 0.01M and NaOH 0.01 M. Higher initial concentration of drugs (5 mg L^−1^ of each) than the typical values found in environmental samples has been selected in the experiments in order to obtain slower kinetics and to facilitate the follow-up of the mineralization. The suspensions were kept in the dark for 30 min, prior to illumination, to reach adsorption equilibrium onto the semiconductor surface. Samples were taken at different time intervals, they were filtered on syringe nylon membrane filters (0.22 μm pore-size) and they were analyzed by LC-MS, as described in the following sections.

### 3.6. Analytical Procedures

#### 3.6.1. Kinetic Studies

Drug concentrations were quantified by a HPLC system consisted of a SIL 20A autosampler and a LC-20AB pump both from Shimadzu (Kyoto, Japan). The analytical column used was a C_18_, 150 × 4.6 mm with 3.5 μm particle size (Pathfinder, Shimadzu Scientific Instruments, Columbia, SC, USA) thermostated at 40 °C. The detector used was SPD 20A DAD coupled in a series with the LC-MS 2010EV mass selective detector, equipped with an atmospheric pressure electrospray ionization source (ESI) in a negative ionization (ΝΙ) and positive ionization (PΙ) mode. A mixture of LC-MS grade water −0.1% formic acid and methanol with a flow rate of 0.4 mL min^−1^ was used as eluent. The time program of the binary mobile phase was: initially 40% methanol, after 5 min changed to 95% methanol and kept stable for 5 min, finally returned to 40% methanol at 12 min. The injection volume was 20 μL and the total run analysis lasted 15 min. The drying gas was operated at flow 10 L min^−1^ at 200 °C. The nebulizing pressure was 100 psi, the capillary voltage −3500 V for negative ionization and 4500 V for positive ionization, while the fragmentation voltage was set at 1.65 V. DCF and ACM were detected at PΙ mode, with retention time 10.23 and 3.97 min and 296 and 151 *m*/*z* precursor molecular ions, respectively. IBU was detected at ΝΙ mode at 11.43 min retention time and 205 *m*/*z* precursor molecular ion, respectively.

#### 3.6.2. By-Product Evaluation

The intermediates formed during the drug mixture photodegradation were characterized by an LTQ Orbitrap Discovery MS high-resolution system operating in the positive and negative ionization mode. The chromatographic analysis was run on an Acquality UPLC HSS T3 column (100 × 2.1 mm, 1.8 μm, Waters, Milford, MA, USA) maintained at a constant temperature of 40 °C. Water (phase A) and methanol (phase B) was used as mobile phases at a flow rate of 500 L min^−1^. A linear gradient progressed from 90% A (initial conditions) to 0% A in 14 min, followed by a linear gradient to 90% A in 20 min. The ESI-source parameters were as follows: Sheath and auxiliary gas flow rate 40 and eight (nitrogen, arbitrary units), respectively; source voltage at 3.60 kV; capillary temperature was maintained at 320 °C. For the fragmentation study, the voltage of the HCD collision cell was set at 40 eV. A resolving power of 30,000 was applied and the Orbitrap mass analyzer was externally calibrated (mass accuracy within ±5 ppm). Chemical compositions and accurate masses of the protonated molecules and their fragments were calculated by Xcalibur software (ThermoFisher Scientific, Waltham, MA, USA).

#### 3.6.3. Mineralization Studies

In order to evaluate the efficiency of the photocatalytic procedure, total organic carbon (TOC) on filtered suspensions (0.22 μm), was evaluated by a Shimadzu TOC V-csh Analyzer equipped with a non-dispersive infrared detector. Ion chromatography (Metrohm) was also used, equipped with an automatic sampler was used for the determination of NO_2_^−^, NO_3_^−^, and Cl^−^ ions. The analytical column used was a Metrosep A Supp 4 (250 × 4.0 mm) with 9 μm particle size. The eluent used was Na_2_CO_3_ 1.8 mM-NaHCO_3_ 1.7 mM with a flow rate of 1 mL min^−1^. The total run analysis lasted 20 min, while the injection volume was 20 μL.

## 4. Conclusions

Effective photocatalysts have been prepared using biobased poly(ethylene furanoate) polymer and high concentrations of TiO_2_ nanoparticles. As was found from SEM-EDX analysis, the dispersion of nanoparticles was fine inside the polymer matrix and its thermal stability was substantially enhanced. The crystal structure of PEF was not affected from TiO_2_ addition due to the lack of interactions between inorganic nanoparticles and organic polymer matrix. Photodegradation experiments were also conducted for a mixture commonly found in wastewaters, anti-inflammatory/analgesic drug mixture. The parameters affecting the photodegradation procedure were optimized for the most efficient synthesized photocatalyst (20% PEF/TiO_2_) and mineralization study was conducted. TOC removal reaches 50% decrease after 360 min of photocatalytic procedure, demonstrating that the photocatalytic treatment needs prolonged irradiation times for leading to complete mineralization. Finally, the TPs generated during the procedure were identified by high-resolution mass spectrometry and their evolution profiles were given. Reutilization experiments proved that the 20% PEF/TiO_2_ have good stability and reusability. The present study provides some important new insights for the use of PEF/TiO_2_ materials as promising alternatives to powder TiO_2_ catalysts for the degradation of organic contaminants.

## Figures and Tables

**Table 1:** DSC data (ΔH^0^_m_ = 137 J/g [42]), T_i_ (initiation of degradation temperature), T_max_ (temperature where degradation occurs on the fastest rate) and solid residue of PEF and its nanocomposites with TiO_2_.

Sample	T_m_ (^o^C)	T_g_ (^o^C)	ΔH_m_ (J/g)	X_c_ (%)	T_i_ (^o^C)	T_max_ (^o^C)	Residue at 550 °C (%)
PEF	213.8	88.0	46.17	33.70	390.3	416.2	7.0
PEF/TiO_2_ 5%	213.8	88.3	45.91	33.51	385.0	424.8	14.6
PEF/TiO_2_ 10%	211.4	88.4	44.98	32.83	384.7	426.7	17.8
PEF/TiO_2_ 15%	211.3	89.1	38.63	28.20	397.0	426.0	28.5
PEF/TiO_2_ 20%	211.5	90.2	30.21	22.05	406.2	433.3	31.2

**Table 2:** High resolution mass spectra data for IBU, DCF, and ACM and their identified TPs derived from mass spectrometric analysis.

t*_R_* (min)	Code Name	Pseudo-Molecular Ion Formula	Theoretical *m*/*z* [m − H]^−^	Experimental *m*/*z* [m − H]^−^	RDBE	Δ (ppm)
14.41	IBU	C_13_ H_17_ O_2_	205.1229	205.1233	5.5	4.844
12.56	I-TP1	C_13_ H_17_ O_3_	221.1178	221.1183	5.5	4.880
12.50	I-TP2	C_12_ H_15_ O_2_	191.1072	191.1078	5.5	5.985
12.02	I-TP3	C_12_ H_15_ O	175.1123	175.1129	5.5	6.615
9.45	I-TP4	C_9_ H_9_ O	133.0653	133.0660	5.5	9.082
8.87	I-TP5	C_9_ H_9_ O_2_	149.0603	149.0610	5.5	8.681
**t_*R*_** **(min)**	**Code Name**	**Pseudo-Molecular Ion Formula**	**Theoretical** ***m*** **/*z* [m + H]^+^**	**Experimental** ***m*** **/*z* [m + H]^+^**	**RDBE**	**Δ** **(ppm)**
14.26	DCF	C_14_H_12_C_l2_NO_2_	296.0245	296.0233	8.5	−2.231
12.38	D-TP1a	C_14_ H_12_ O_3_ N Cl_2_	311.0116	312.0180	8.5	−2.805
12.96	D-TP1b	C_14_ H_12_ O_3_ N Cl_2_	311.0116	312.0180	8.5	−2.805
8.81	D-TP2	C_6_ H_6_ O N Cl_2_	177.9825	177.9812	3.5	−5.033
16.84	D-TP3	C_14_H_10_O_3_NCl_2_	310.0038	310.0026	9.5	−2.016
6.15	D-TP4	C_14_H_12_O_4_ NCl_2_	328.0138	328.0132	8.5	−1.798
15.96	D-TP5	C_14_H_10_O_2_NCl_2_	294.0089	294.0072	9.5	−3.777
11.98	D-TP6	C_13_H_10_ONCl_2_	266.0167	266.0123	8.5	−4.120
14.02	D-TP7	C_13_H_10_O_2_NCl_2_	282.0062	282.0074	8.5	−3.229
12.70	D-TP8	C_13_H_8_Cl_2_NO_2_	280.0013	280.0055	9	0.941
5.18	ACM	C_8_ H_10_ O_2_ N	152.0712	152.0696	4.5	−6.609
4.48	A-TP1a	C_8_ H_10_ O_3_ N	168.0661	168.0647	4.5	−4.877
4.70	A-TP1b	C_8_ H_10_ O_3_ N	168.0661	168.0647	4.5	−4.877
3.30	A-TP2a	C_8_ H_10_ O_4_ N	184.0610	184.0598	4.5	−3.446
3.80	A-TP2b	C_8_ H_10_ O_4_ N	184.0610	184.0598	4.5	−3.446
3.98	A-TP2c	C_8_ H_10_ O_4_ N	184.0610	184.0598	4.5	−3.446
4.65	A-TP2d	C_8_ H_10_ O_4_ N	184.0610	184.0598	4.5	−3.446

## Supplementary Materials

The following are available online at http://www.mdpi.com/1420-3049/24/3/564/s1, Figure S1: Effect of drug concentration (C(PEF/TiO2 20 wt% = 600 mg L^−1^); (a) ACM; (b) IBU; (c) DCF.; Figure S2: The effect PEF/TiO_2_ 20 wt% loading (C_0_(drug) = 5 mg L^-1^); (a) ACM; (b) IBU; (c) DCF; Figure S3: Effect of pH, during photocatalytic treatment (C_o_(drug) = 5 mg L^−1^, C((PEF/TiO_2_ 20 wt%) = 600 mg L^−^); (a) ACM; (b) IBU; (c) DCF.
