# Supplementary material for: Biobased Poly(ethylene furanoate) Polyester/TiO2 Supported Nanocomposites as Effective Photocatalysts for Anti-inflammatory/Analgesic Drugs
Source: Molecules. 2019 Feb 4;24(3):564. doi: 10.3390/molecules24030564 (PMC6384769; doi:10.3390/molecules24030564)
Supplement: Supplementary file 1 [file molecules-24-00564-s001.pdf]

Supplementary data: Biobased poly(ethylene furanoate) polyester/TiO2 supported nanocomposites as effective photocatalysts for anti-inflammatory/analgesic drugs

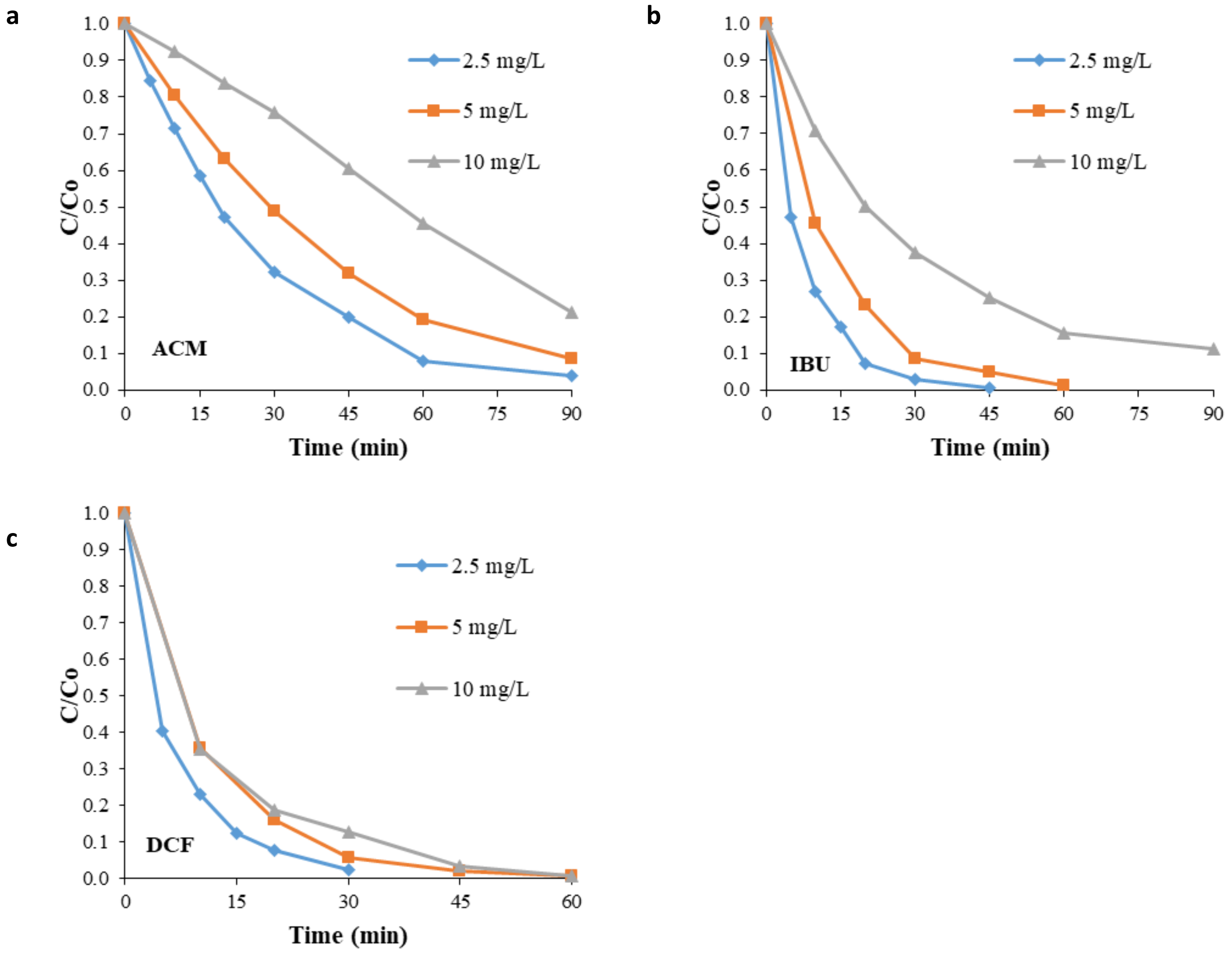


S1. Effect of drug concentration (C(PEF/$TiO_2$ 20 wt% =600 mg $L^{-1}$); (a)ACM; (b) IBU; (c) DCF.

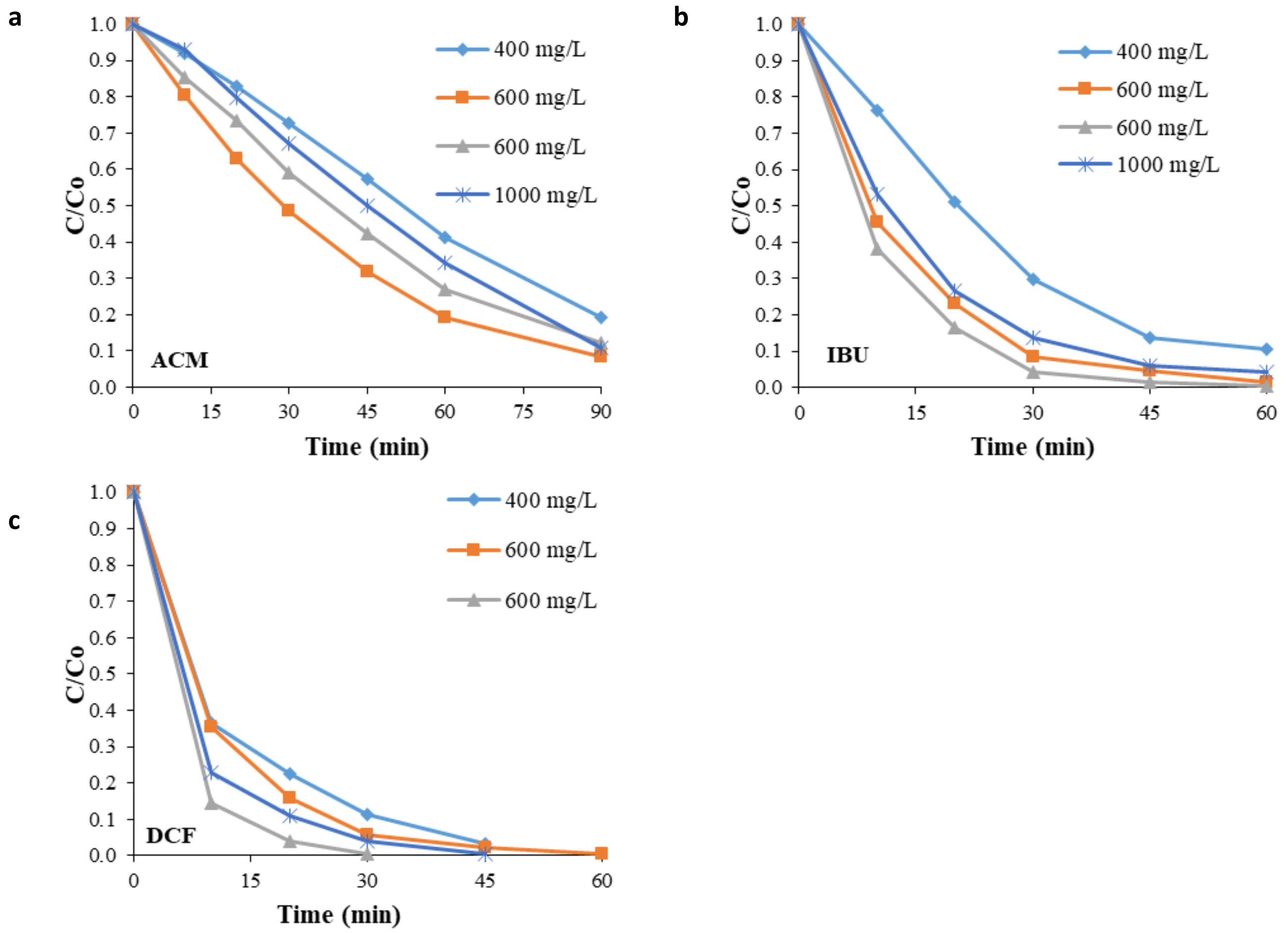


S2. The effect PEF/$TiO_2$ 20 wt% loading ($C_0$(drug)=5 mg $L^{-1}$); (a)ACM; (b) IBU; (c) DCF.

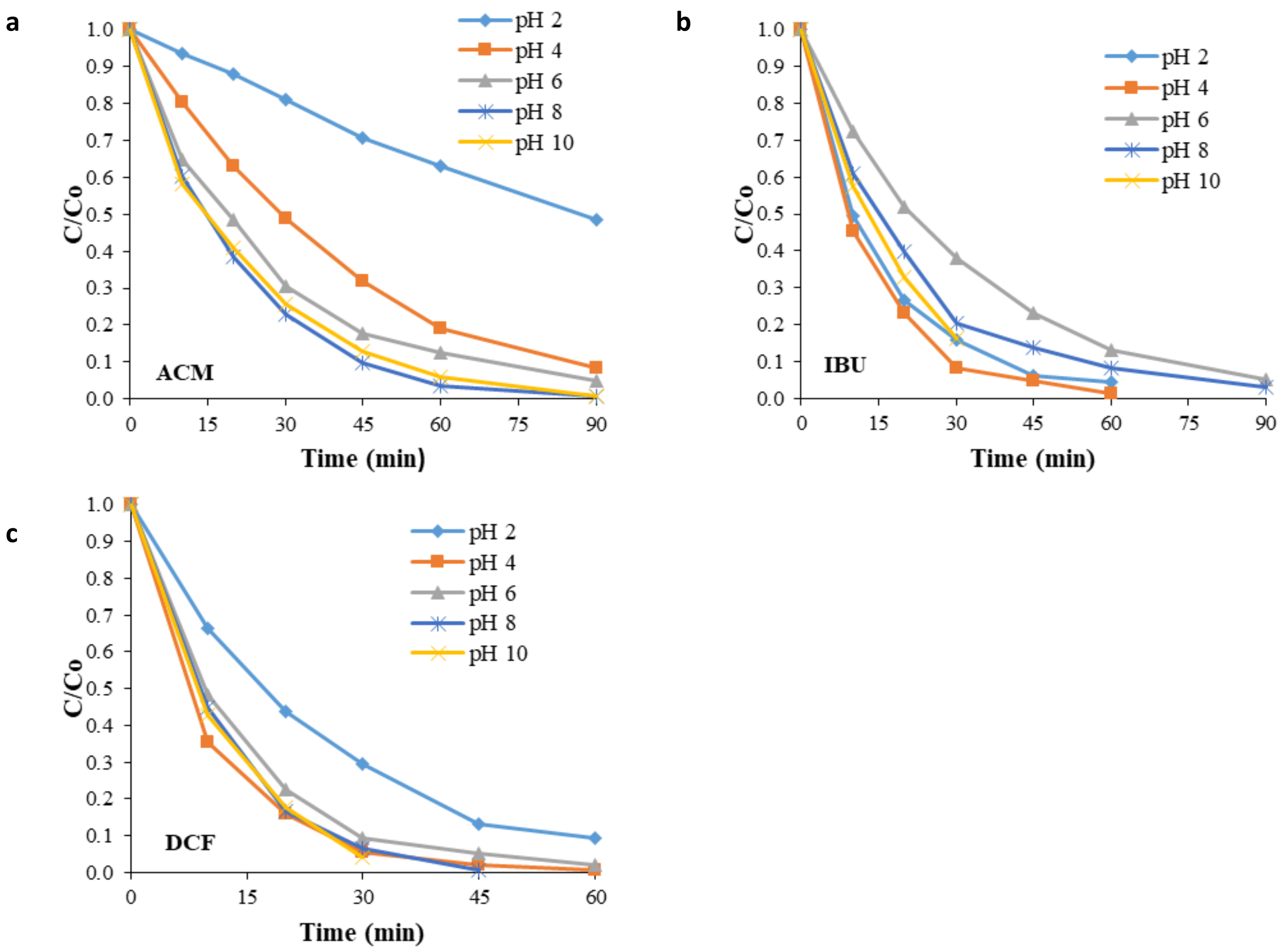


S3. Effect of pH, during photocatalytic treatment ($C_o$(drug)=5 mg $L^{-1}$, C((PEF/$TiO_2$ 20wt %)=600 mg $L^{-1}$); (a)ACM; (b) IBU; (c) DCF.
